# Significance testing as perverse probabilistic reasoning

**DOI:** 10.1186/1741-7015-9-20

**Published:** 2011-02-28

**Authors:** M Brandon Westover, Kenneth D Westover, Matt T Bianchi

**Affiliations:** 1 Department of Neurology, Massachusetts General Hospital, Harvard Medical School, Boston, MA, USA; 2 Harvard Radiation Oncology Program, Harvard Medical School, Boston, MA, USA

## Abstract

Truth claims in the medical literature rely heavily on statistical significance testing. Unfortunately, most physicians misunderstand the underlying probabilistic logic of significance tests and consequently often misinterpret their results. This near-universal misunderstanding is highlighted by means of a simple quiz which we administered to 246 physicians at two major academic hospitals, on which the proportion of incorrect responses exceeded 90%. A solid understanding of the fundamental concepts of probability theory is becoming essential to the rational interpretation of medical information. This essay provides a technically sound review of these concepts that is accessible to a medical audience. We also briefly review the debate in the cognitive sciences regarding physicians' aptitude for probabilistic inference.

## Background

Medicine is a science of uncertainty and an art of probability. - Sir William Osler [1]

While probabilistic considerations have always been fundamental to medical reasoning, formal probabilistic arguments have only become ubiquitous in the medical literature in recent decades [2,3]. Meanwhile, many have voiced concerns that physicians generally misunderstand probabilistic concepts, with potential serious negative implications for the quality of medical science and ultimately public health [3-12]. This problem has been demonstrated previously by surveys similar to the following quiz [13], which we administered to a group of 246 physicians at three major US teaching hospitals (Barnes Jewish Hospital, Brigham and Women's Hospital, and Massachusetts General Hospital). The reader is likewise invited to answer before proceeding.

Consider a typical medical research study, for example designed to test the efficacy of a drug, in which a null hypothesis *H*_0 _('no effect') is tested against an alternative hypothesis *H*_1 _('some effect'). Suppose that the study results pass a test of statistical significance (that is *P*-value <0.05) in favor of *H*_1_. What has been shown?

1. *H*_0 _is false.

2. *H*_1 _is true.

3. *H*_0 _is probably false.

4. *H*_1 _is probably true.

5. Both (1) and (2).

6. Both (3) and (4).

7. None of the above.

The answer profile for our participants is shown in Table 1. This essay is for readers who, like 93% of our respondents, did not confidently select the correct answer, (7), 'None of the above'. We hasten to assure the reader that this is not a trick question. Rather, it is a matter of elementary probabilistic logic. As will be clear by the end of this essay answers (1) to (6) involve 'leaping to conclusions', in violation of the basic law of probabilistic inference, Bayes' rule. We will see that Bayes' rule is an essential principle governing all reasoning in the face of uncertainty. Moreover, understanding Bayes' rule serves as a potent prophylaxis against statistical fallacies such as those underlying the apparent plausibility of the six erroneous answers in this little quiz.

**Table 1:** Quiz answer profile.

Answer	(1)	(2)	(3)	(4)	(5)	(6)	(7)
Number	8	0	58	37	6	69	12
Percent	4.2	0	30.5	19.5	3.2	36.3	6.3

Despite its central place in the theory of probabilistic inference, Bayes' rule has been largely displaced in the practice of quantitative medical reasoning (and indeed in the biological and social sciences generally) by a statistical procedure known as 'significance testing'. While significance testing can, when properly understood, be seen as an internally coherent aid to scientific data analysis [14], it is usually misunderstood as a way to bypass Bayes' rule, which we shall see is a perversion of probabilistic reasoning. Embarrassingly, fallacious uses of significance testing continue to flourish despite being under constant criticism in the statistical literature since its inception in the 1960 s [5,13,15-17]. The reasons for this state of affairs derive from a complex web of social and philosophical factors. However, we believe a more immediate barrier to physicians understanding probability theory is the lack of adequate literature explaining the subject in a way that physicians can relate to. Therefore, we have written this essay with three aims in mind. The first aim, addressed in 'Discussion, Part I', is to explain the basic concepts of probability theory to physicians, and in particular to provide a detailed account of the 'origin', mechanics, and meaning of Bayes' rule. The second aim, covered in 'Discussion, Part II', is to provide an accurate technical explanation of the two ingredients of significance testing: binary hypothesis testing and *P*-values. Finally, we aim to show how understanding Bayes' rule protects against common errors of statistical reasoning, such as those involved in choosing the wrong answers to our introductory quiz.

## Discussion, Part I: probability in medicine

### Reasoning under uncertainty

They say that Understanding ought to work by the rules of right reason. These rules are, or ought to be, contained in Logic; but the actual science of logic is conversant at present only with things either certain, impossible, or entirely doubtful, none of which (fortunately) we have to reason on. Therefore the true logic for this world is the calculus of Probabilities, which takes account of the magnitude of the probability which is, or ought to be, in a reasonable man's mind. - James Clerk Maxwell [18]

#### The inadequacy of deductive logic

Since Aristotle the mainstream Western view has been that rationality means reasoning according to the rules of deductive logic [19,20]. The basic building block of deductive logic is the syllogism, for example:

$$\begin{array}{l} \text{if}\;A\;\text{is}\;\text{true,}\;\text{then}\;B\;\text{is}\;\text{true}\text{.}\\ A\;\text{is}\;\text{true}\text{.}\\ ∴B\;\text{is}\;\text{true}\text{.} \end{array}$$

Or, similarly:

$$\begin{array}{l} \text{if}\;A\;\text{is}\;\text{true,}\;\text{then}\;B\;\text{is}\;\text{true}\text{.}\\ B\;\text{is}\;\text{false}\text{.}\\ ∴A\;\text{is}\;\text{false}\text{.} \end{array}$$

These logical forms play a role in straightforward medical diagnostic scenarios like the following:

• 75 year old man with fever, productive cough, chest x-ray showing consolidation of the right upper lobe, sputum culture positive for gram positive cocci in clusters.

Diagnosis: Pneumonia.

• 50 year old previously healthy man with sudden onset painful arthritis of the MTP joint of his right great toe, arthrocentesis positive for needle-shaped, negatively birefringent crystals.

Diagnosis: Gout.

The reasoning required to make these diagnoses is essentially syllogistic, that is a matter of checking that the definitions of the disorders are satisfied, then drawing the inevitable conclusion.

However, medical reasoning frequently requires going beyond syllogistic reasoning. For example, consider the following argument type:

$$\begin{array}{l} \text{if}\;A\;\text{is}\;\text{true,}\;\text{then}\;B\;\text{is}\;\text{true}\text{.}\\ B\;\text{is}\;\text{true}\text{.}\\ ∴A\;\text{becomes}\;\text{more}\;\text{plausible}\text{.} \end{array}$$

Of course, given the premise (*A *⇒ *B*), the truth of *B *does not, strictly speaking, imply the truth of *A*, hence the use of the term 'plausible' to denote an implication that falls short of certitude. Arguments of this kind, which have been aptly called 'weak syllogisms' [21], are indispensable in everyday medical reasoning. For example, it is reasonable to assert that patients with appendicitis will have abdominal pain, and we accept abdominal pain as grounds for suspecting appendicitis, though logically there are numerous other possible explanations for abdominal pain. In a similar vein, consider these additional typical case vignettes and possible diagnoses:

• 45 year old homeless alcoholic man brought in by police with confusion, disorderly behavior, and breath smelling of alcohol. Diagnosis: Ethanol intoxication.

• 75 year old nursing home resident with known heart failure presents with confusion and shortness of breath. Physical examination reveals rales, 3+ lower extremity pitting edema, labored breathing. Diagnosis: CHF exacerbation.

• 55 year old male presents to ED with acute onset substernal chest pain. Diagnosis: Gastric reflux.

Most physicians quickly assign rough degrees of plausibility to these diagnoses. However, in these cases it is reasonable to entertain alternative diagnoses, for example in the first case other intoxicants, or meningitis; and in the second case pulmonary embolus, pneumonia, or myocardial infarction. In the third case the stated diagnosis is only weakly plausible, and most physicians would doubt it at least until other possibilities (for example myocardial ischemia) are ruled out. In each case, there is insufficient information to make a certain (that is logically deductive) diagnosis; nevertheless, we are accustomed to making judgements of plausibility.

Stepping back once more, we can add to the list of argument types frequently needed in medical reasoning the following additional examples of even weaker 'weak syllogisms':

$$\begin{array}{l} \text{If}\;A\;\text{is}\;\text{true,}\;\text{then}\;B\;\text{becomes}\;\text{more}\;\text{plausible}\text{.}\\ B\;\text{is}\;\text{true}\text{.}\\ ∴A\;\text{becomes}\;\text{more}\;\text{plaussible}\text{.} \end{array}$$

and

$$\begin{array}{l} \text{If}\;A\;\text{is}\;\text{true,}\;\text{then}\;B\;\text{becomes}\;\text{more}\;\text{plausible}\text{.}\\ B\;\text{is}\;\text{plausible}\text{.}\\ ∴A\;\text{becomes}\;\text{more}\;\text{plaussible}\text{.} \end{array}$$

As in syllogistic reasoning, weak syllogistic reasoning combines prior knowledge (for example knowledge of medicine and clinical experience) with new data (for example from seeing patients, lab tests, or new literature), but the knowledge, data, and conclusions involved lack the certainty required for deductive logical reasoning. The practice of formulating differential diagnoses, and the fact that physicians do not routinely test for every possibility in the differential, shows that physicians do in fact routinely assign degrees of plausibility. The same can be said of most situations in everyday life, in which the ability to judge which possibilities to ignore, which to entertain, and how much plausibility to assign to each constitute 'common sense'. We now explore the rules that govern quantitative reasoning under uncertainty.

### Cox's theorem and the laws of plausible reasoning

There is only one consistent model of common sense. - ET Jaynes [21]

How might one go about making the 'weak syllogisms', introduced above, into precise quantitative statements? Let us attempt to replace the loose statement that '*A *becomes more plausible in light of *B*', with a formula telling us how plausible *A *has become. For this purpose, let us denote by *A *and *B *the propositions '*A *is true' and '*B *is true'. We assume that we have already assigned an 'a priori' value to the plausibility of *A*, denoted P(A).. We wish to quantify how much more plausible *A *becomes once we learn the additional information given in the premises, comprising the plausibility of *B*, denoted P(B), and the plausibility of *B *when *A *is true, P(B|A). We focus on the third and 'weakest' syllogism, of which the other weak syllogisms are special cases. A quantitative re-writing of this statement takes the following form:

$$\begin{array}{l} \text{The}\;\text{plausibility}\;\text{of}\;A\;(\text{without}\;\text{regard}\;\text{to}\;B)\;\text{is}\;\text{equal}\;\text{to}\;P(A).\\ \text{The}\;\text{plausibility}\;\text{of}\;B\;(\text{without}\;\text{regard}\;\text{to}\;A)\;\text{is}\;\text{equal}\;\text{to}\;P(B).\\ \text{The}\;\text{plausibility}\;\text{of}\;B\;\text{when}\;A\;\text{is}\;\text{true is}\;\text{equal}\;\text{to}\;P(B|A).\\ ∴\text{The}\;\text{plausibility}\;\text{of}\;A\;\text{when}\;B\;\text{is}\;\text{true is}\;\text{equal}\;\text{to}\;P(A|B). \end{array}$$

From this it is apparent that what we are seeking is a formula that gives the strength of the conclusion as a function, *f*, of the quantities involved in the premises, that is an equation of the form:

P(A|B)=f(P(A),P(B),P(B|A)).

RT Cox (1898-1991) [22] and ET Jaynes (1922-1998) [23] were able to prove mathematically that the only possible formula of this form suitable for measuring plausibilities was in fact:

$$Pr(A|B)=\frac{Pr(B|A)Pr(A)}{Pr(B)},$$

where the numbers denoted by *Pr *represent probabilities, subject to the basic laws of probability theory, which are:

• 0 ≤ *Pr*(*A*) ≤ 1,

• *Pr*(*A*) = 0 when *A *is known to be false,

• *Pr*(*A*) = 1 when *A *is known to be true,

• $Pr(A)\;+Pr(\bar{A})=1$,

• $Pr(B)=Pr(B,A)+Pr(B,\bar{A})$

where *Pr*(*A*, *B*) represents the probability that propositions *A *and *B *are both true. In other words, this result, known as 'Cox's theorem', proved that the only acceptable way to quantify plausibilities P is to use probabilities, *Pr*, and that the central rule involved in considerations of plausibility is the formula for computing conditional probabilities, Bayes' rule. Readers interested in a more complete account of Cox's theorem are referred the excellent discussions by Jaynes [23] and more recently by Van Horn [24]. *A *brief review of the interpretation of each of the basic laws of probability theory, using Venn diagrams, can be found in the Additional file 1.

In the rest of the paper, we will use the more common form for Bayes' rule, which is derived from the form given above by simple substitutions using the basic relations of probability just cited:

$$Pr(A|B)=\frac{Pr(B|A)Pr(A)}{Pr(A)Pr(B|A)+Pr(\bar{A})Pr(B|\bar{A})}.$$

This form is useful in that it makes explicit the fact that Bayes' rule involves three distinct ingredients, namely *Pr*(*A*), (and its converse $Pr(\bar{A})=1-Pr(A))$, *Pr*(*B*|*A*), and $Pr(B|\bar{A})$. The meanings of these ingredients will become clear in the next section.

We pause before proceeding to comment on our focus in this essay on simple applications of Bayes' rule. Our aim is to explain the basic concepts governing probabilistic inference, a goal we believe is best served by using very simple applications of Bayes' rule to evaluating mutually exclusive truth claims (that is 'binary hypotheses'). We hasten to add that binary hypothesis comparison is not necessarily always the best approach. For instance, in the quiz beginning this essay, rather than pitting *H*_0 _('no effect') against hypothesis *H*_1 _('some effect'), it may be more informative to consider a range of possible value for the strength of the effect, and to compute a probability distribution over this range of possible effect sizes, from which we could also 'read off' the credibility of the hypothesis that the effect size is equal to or close to zero. The perils of inappropriate uses of binary hypothesis testing, and alternative Bayesian methods for assessing hypotheses, are discussed at length in several good books and articles, for example [25,26].

Indeed, much real-world medical reasoning cannot be naturally reduced to evaluating simple 'true/false' judgements, but requires instead the simultaneous analysis of multiple data variables, which often take on multiple or a continuous range of values (not just binary). There are frequently not just two but many competing interpretations of medical data. Moreover, we are often more interested in inferring the magnitude of a quantity or strength of an effect rather than simply whether a statement is true or false. Similarly, evaluating medical research typically involves reasoning too rich to be naturally modeled as binary hypothesis testing (contrary to the spirit of Fisher's famous pronouncement that 'every experiment may be said to exist only in order to give the facts a chance of disproving the null hypothesis' [27]). Similar points can be made about the richness of the inference characteristically required in much of everyday life. In principle, and increasingly in practice, these complex situations in fact can be given an appropriate quantitative probabilistic (that is 'Bayesian') analysis. Accordingly, we wish to make the reader aware that there exists large and expanding literature, built upon the foundation of Bayes' rule, which goes far beyond the simple considerations of binary hypothesis testing discussed here. To give just a few examples, Bayes' rule is the basis for: sophisticated methods for the rational analysis of complex data [26,28,29], especially data from medical clinical trials [30-36]; probabilistic models in cognitive science of sensory perception, learning, and cognition [20,37-42]; and increasingly successful approaches to real-world problems in artificial intelligence including search engine technology, general pattern recognition in rich data sets, computer vision and speech recognition, terrorist threat surveillance, and early detection of disease outbreaks [19,43-56].

Nevertheless, understanding the ongoing work at the frontiers of modern probability theory requires first a sound understanding of Bayes' rule in its most elementary form, the focus of this essay.

### The 'subjective' interpretation of probability

It is important to appreciate that the interpretation of mathematical probability as a measure of plausibility, that is as a 'degree of belief', is not the only way of conceptualizing probability. Indeed, in mathematics probability theory is usually developed axiomatically, starting with the rules of probability as 'given' [57]. Probability theory can also be developed from a 'frequentist' point of view, with probabilities interpreted as the fraction of events for which a particular proposition is true in series of cases over time, or within a collection or population of cases. The frequentist view has some obvious limitations in that it does not strictly allow one to talk about the probability of particular events, for example the probability that Mr. Jones has pneumonia. However, in practice the views are not incompatible: If we know nothing else about Mr. Jones, it may be reasonable to set one's initial assignment of the probability that Mr. Jones has pneumonia equal to the fraction of persons in similar circumstances who were ultimately found to have pneumonia.

The interpretation of probabilities as degrees of belief is often called the 'subjective interpretation of probability,' or more succinctly, 'Bayesian probability,' because Thomas Bayes is credited as the first to develop a coherent way to estimate probabilities of single events [58]. There is a long history of tension between the frequentist and Bayesian interpretations of probability. However, this controversy has waned, in part because of Cox's theorem, but also because of the explosion in the number of practical applications of Bayes' rule that have become possible since the computer revolution [19,20,53,59,60].

### The three ingredients of Bayes' rule

An intuition for why Bayes' rule has the form that it does can be gained by observing the effects produced by changing the values of each of its three variables. For concreteness, we frame our discussion in terms of the problem of distinguishing appendicitis from other causes of abdominal pain in a pediatric emergency department on the basis of the presence or absence of fever. In this example, fever is taken as evidence of appendicitis, so we have the following labels for the four possible combinations of fever (*F*) and appendicitis (*A*): (*F, A*) = 'true positives', (F¯,A¯)='true negatives', (F,A¯)='false positives', and (F¯,A)='false negatives'. We note that Bayes' rule combines three essential ingredients: the prior probability of appendicitis *Pr*(*A*) (and its converse $Pr(\bar{A})=1-Pr(A))$ and the two conditional probabilities *Pr*(*F*|*A*) and $Pr(F|\bar{A})$, which we will call the true positive and false positive rates, respectively.

### Anatomy of Bayes' rule

The importance of each of the ingredients of Bayes' rule, the three arguments *Pr*(*A|F*) = *f*(*a, b, c*), where *a *= *Pr*(*A*), *b *= *Pr*(*F|A*), and $c=Pr(F|\text{​}\text{​}\bar{A})$, is most easily grasped by considering extreme cases. We invite the reader to consider the arguments first from the standpoint of 'common sense' before checking that the conclusion is indeed borne out mathematically by Bayes' rule.

1. Suppose that somehow we know, independent of fever status, that 100% of the patients have appendicitis, *Pr*(*A*) = 1. In this case, fever can have no effect on the probability of appendicitis, that is *Pr*(*A|F*) must be equal to *Pr*(*A*), regardless of the other two factors *Pr*(*F|A*) and $Pr(F|\bar{A})$. Thus *Pr*(*A|F*) must depend on the prior probability, *Pr*(*A*).

2. Next, suppose every child with appendicitis has a fever, *Pr*(*F|A*) = 1, and every child without appendicitis is afebrile, $Pr(F|\bar{A})=0$. Then knowing the child's temperature would be equivalent to knowing the diagnosis. Thus, *Pr*(*A|F*) must be equal to one, and $Pr\text{(}A|\text{​}\bar{F}\text{)}$ must equal zero, regardless of *Pr*(*A*). Thus, *Pr*(*A|F*) must depend on some combination of the true positive rate, *Pr*(*F|A*), and false positive rate, $Pr(F|\bar{A})$ respectively.

3. To see that *Pr*(*F|A*) and $Pr(F|\bar{A})$ can in fact act as independent variables in affecting *Pr*(*A|F*), for the next two cases, let our uncertainty before taking the child's temperature be maximal, $Pr(A)=Pr(\bar{A})=1/2$. Now suppose that all patients with appendicitis have fever, *Pr*(*F|A*) = 1. Then the predictive value of fever as a marker of appendicitis must vary inversely with the frequency of fever in patients without appendicitis, $Pr(F|\bar{A})$ (or equivalently, monotonically with the specificity $Pr(\bar{F}|\bar{A})$). Thus, *Pr*(*A|F*) must depend on the true positive rate, *Pr*(*F|A*).

4. Suppose that no one with appendicitis gets fevers, *Pr*(*F|A*) = 0. Then the presence of fever automatically rules out appendicitis, regardless of any other information. Thus, *Pr*(*A|F*) must depend on the false positive rate, $Pr(F|\bar{A})$.

These arguments show that the formula for the 'posterior probability', that is the probability of appendicitis given fever, *Pr*(*A\F*), must take into account all three quantities, *Pr*(*A*), *Pr*(*F|A*), and $Pr(F|\bar{A})$, as indeed Bayes' rule does.

### Physiology of Bayes' rule

We now explore how the output of Bayes' rule varies with its three inputs. Interactive online computer programs may also be helpful for gaining intuition, and can be found using the following references: [61,62].

Consider a hypothetical population of 1,000 patients evaluated for abdominal pain in the pediatric emergency room, some with fever, some with appendicitis, some with both, and some with neither. We will systematically vary the proportions of each subpopulation and observe the output of Bayes' rule. The numbers used in these examples are summarized in Table 2.

**Table 2:** Hypothetical statistics for fever and appendicitis.

***TP***	|	***FP***			***Pr*(*F*|*A*)**	|	***Pr*(*A*)**
			
***FN***	|	***TN***			$Pr(F|\bar{A})$	|	***Pr***(***A***|***F***)


62	|	112			56%	|	11%
			
49	|	777			13%	|	36%


79	|	112			71%	|	11%
			
32	|	777			13%	|	41%


45	|	112			40%	|	11%
			
66	|	777			13%	|	29%


62	|	136			56%	|	11%
			
49	|	753			15%	|	31%


62	|	88			56%	|	11%
			
49	|	801			10%	|	41%


139	|	192			56%	|	45%
			
111	|	558			26%	|	42%


22	|	121			56%	|	4%
			
18	|	839			13%	|	6%

Initially, suppose that among our 1,000 patients, 121 are ultimately found to have appendicitis. Fever was present on initial presentation in 174 patients, of which 62 are found to have appendicitis. The number of true positives, false positives, false negatives, and true negatives calculated from these numbers are listed in the first row of Table 2. In turn, we estimate the sensitivity (also known as true positive rate) of fever as a sign for appendicitis as:

Pr=(F|A)=TP/(TP+FN)=62/(111)=56%,

the false positive rate (also known as 1-specificity) as:

$$Pr(F|\bar{A})=FP/(FP+TN)=112/889=13\%,$$

and the prior probability (also known as prevalence) as:

Pr(A)=(TP+FN)/(TP+FP+FN+TN)=112/1,000=11%.

**Figure 1:**
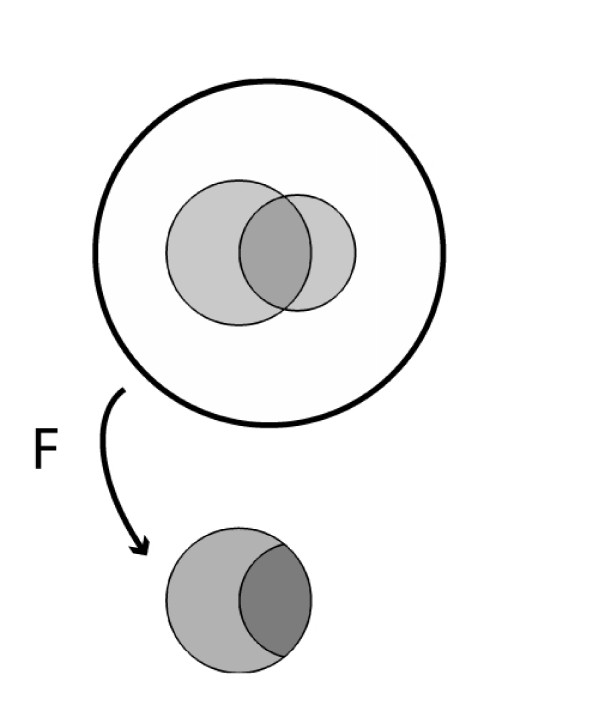
**Reference population of patients with appendicitis and fever, showing the result of conditioning on the presence of fever**.

This situation is shown schematically in Figure 1 in which the area enclosed by the outer circle represents the entire patient population; the larger internal shaded region represents the number of patients with fever; the smaller internal shaded region represents the number of patients with appendicitis; and the area of overlap represents the number of patients with both appendicitis and fever.

So, in a febrile child complaining of abdominal pain, what is the probability of appendicitis? Based on the information above, most physicians give an answer close to 56%, a conclusion reached apparently by mentally replacing the prior probability *Pr*(*A*) with the true positive rate *Pr*(*F|A*), thus confusing the latter with the correct quantity, *Pr*(*A|F*) [11,12,61]. The correct answer is computed by taking the fraction of patients with appendicitis among those with fever, *Pr*(*A|F*) = *TP/*(*TP + FP*) = 62/174 = 36%. Figure 1 illustrates this calculation graphically, where the act of taking fever as 'given' is depicted as collapsing the population to just those patients who have fever. As expected, finding that a patient with abdominal pain has fever increases the probability of appendicitis - in fact, the probability more than triples (from 11% to 36%) - but, critically, the probability increases from the prior probability *Pr*(*A*). One needs to know the prior probability *Pr*(*A*) to calculate the posterior probability *Pr*(*A|F*).

#### >Varying *Pr(F|A)*

**Figure 2:**
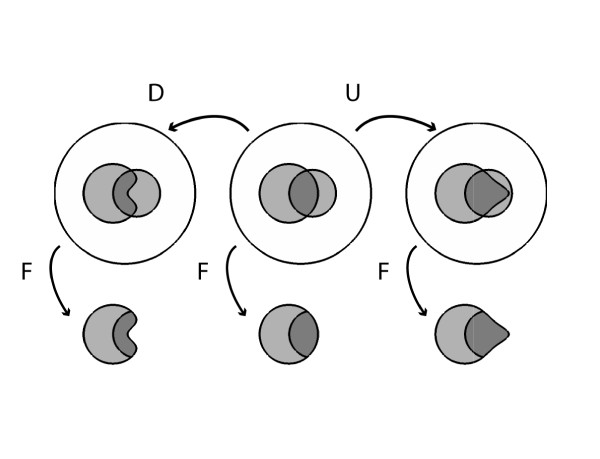
**Effects on posterior probability of changes in sensitivity, while holding prior probability and false positive rate constant**.

Suppose we increase the true positive rate *Pr*(*F|A*) from 56% to 71% (Figure 2). This increases the posterior probability of appendicitis from 36% to 41%. These increases correspond to an increase in the number of appendicitis patients who have fever from 62 to 79, or graphically to a 15% expansion of the part of the fever region that is within the appendicitis region, with the result that a 5% larger fraction of the fever region contains appendicitis. Conversely, a decrease in the true positive rate *Pr*(*F|A*) from 56% to 40% decreases the posterior probability *Pr*(*A|F*) from 36% to 29%. These changes correspond numerically to a decrease in the number of patients with fever and appendicitis from 62 to 45.

#### Varying $Pr(F|\bar{A})$

**Figure 3:**
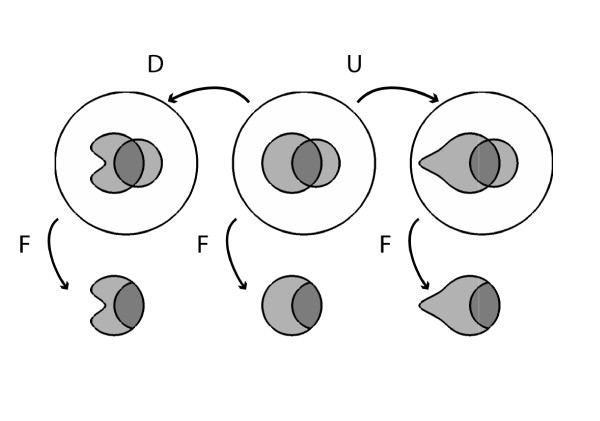
**Effects on posterior probability of changes in false positive rate, while holding prior probability and sensitivity constant**.

Next let us slightly increase the false positive rate $Pr(F|\bar{A})$ from 13% to 15% (Figure 3). This pushes the posterior probability *Pr*(*A|F*) down from 36% to 31%, and corresponds numerically to increasing the number of febrile patients without appendicitis from 112 to 136, or graphically to a 2% growth of the part of the fever region that is outside the appendicitis region, with the result that the fractional area of the fever region covered by appendicitis shrinks by 5%.

Conversely, a decrease in the false positive rate $Pr(F|\bar{A})$ from 13% to 10% pushes the posterior probability *Pr*(*A|F*) up from 36% to 41%. This corresponds numerically to decreasing the number of febrile patients without appendicitis from 112 to 88, or graphically to a shrinkage of the part of the fever region that is outside the appendicitis region by 3%, with the result that the fractional area of the fever region covered by appendicitis expands by 5%.

#### Varying *Pr(A)*

**Figure 4:**
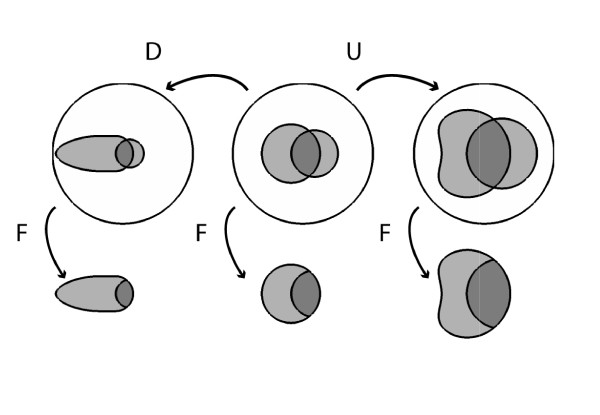
**Effects on posterior probability of changes in prior probability, while holding sensitivity and false positive rate constant**.

Finally, consider increasing the prior probability of appendicitis *Pr*(*A*) from 11% to 25% while holding the true and false positive rates fixed at *Pr*(*F|A*) = 56% and $Pr(F|\bar{A})=26\%$. This change raises the posterior probability *Pr*(*A|F*) from 36% to 42%. In the corresponding Venn diagram shown in Figure 4 increasing *P*(*A*) corresponds to simply increasing the area of *A*; *Pr*(*F|A*) is held fixed by increasing the area of *F *within *A *proportionately, whereas keeping the same value for $Pr(F|\bar{A})$ requires a compensatory shrinkage of the shape for *F*. Likewise, decreasing the prior probability from 11% to 4% lowers the posterior probability from 36% to 16%, which in the accompanying Venn diagram requires shrinking *A*, shrinking the part of *F *within *A *proportionately to hold *Pr*(*F|A*) fixed, and stretching the shape of *F *outside of *A *to maintain the fixed value of $Pr(F|\bar{A})$. The numbers for this example are shown in Table 2 and Figure 4.

### Summary of the general rules

These examples illustrate the following general principles (assuming a 'positive' test result):

• Increasing the true positive rate (sensitivity) pushes the posterior probability upward, whereas decreasing the true positive rate pushes the posterior probability downward.

• Increasing the false positive rate (1-specificity) pushes the posterior probability downward, whereas decreasing the false positive rate pushes the posterior probability upward.

• Increasing the prior probability pushes the posterior probability upward, whereas decreasing the prior probability pushes the posterior probability downward.

**Figure 5:**
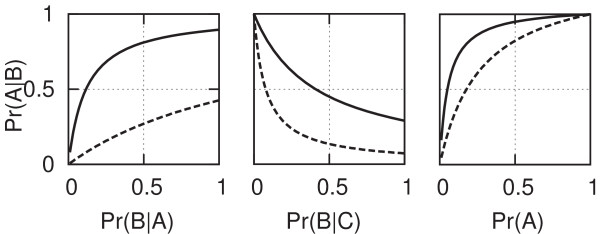
**Illustration of how the posterior probability depends on the three parameters of Bayes' rule**. Each plot shows two curves for the posterior probability as a function of one of the three parameters (with the remaining two parameters held constant) chosen from among one of two sets of values for(*Pr*(*A*), *Pr*(*F*|*A*), $Pr(F|\bar{A})$), either (0.3, 0.95, 0.05) or (0.1, 0.7, 0.15).

We emphasize again that in every case the posterior probability goes up or down from the prior probability, rather than being replaced by any of the three quantities. These general rules are illustrated in the graphs in Figure 5.

### End of Part I

Uncertainty suffuses every aspect of the practice of medicine, hence any adequate model of medical reasoning, normative or descriptive, must extend beyond deductive logic. As was believed for many decades, and recently proven by Cox and Jaynes, the proper extension of logic is in fact probability theory, with Bayes' rule as the central rule of inference. We have attempted to explain in an accessible way why Bayes' rule has its particular form, and how its behaves when its parameters vary. In the Part II, we investigate ways in which probability theory is commonly misunderstood and abused in medical reasoning, especially in interpreting the results of medical research.

## Discussion, Part II: significance testing

Every experiment may be said to exist only in order to give the facts a chance of disproving the null hypothesis. - RA Fisher [27]

Armed with our understanding of the anatomy and physiology of Bayes' rule, we are prepared for pathophysiology. In Part II we explore common misinterpretations and misuses of elementary medical statistics that occur in the application of significance testing, and how these can be effectively treated by applying our understanding of Bayes' rule.

Before one can appreciate the problems with significance testing, one needs a clear understanding of a few concepts from 'classical statistics', namely binary hypothesis testing and *P*-values. We now proceed to review these concepts.

### Binary hypothesis testing

Binary hypothesis testing is familiar to most physicians as the central concept involved in judging the results of clinical trials. The basic setup was encountered in the quiz that began the paper. For any proposition *A*, we set up two hypotheses: *H*_0 _= '*A *is not true', called the null hypothesis; and *H*_1 _= '*A *is true', called the alternative hypothesis. In our quiz, the effect of a new drug was being investigated and we had *H*_0 _= 'the drug has no effect' vs. *H*_1 _= 'the drug has some effect'. One of these statements must be true as a matter of logical necessity. To find out which one, an experiment is carried out (for example a clinical trial), resulting in data *D*. We then conclude, through a procedure described below, that the data either favors *H*_0_, called 'affirming the null hypothesis,' or favors *H*_1_, called 'rejecting the null hypothesis.' We will denote our conclusions as either *D*_0 _= 'the data favor the null hypothesis', or *D*_1 _= 'the data favor the alternative hypothesis'.

Our conclusions can be right or wrong in four ways (see Table 2). Correct results include 'true positives' (concluding *D*_1 _when *H*_1 _is true), and 'true negatives' (concluding *D*_0 _when *H*_0 _is true); the corresponding probabilities *Pr*(*D*_0_*|H*_0_) and *Pr*(*D*_1_*|H*_1_) are called the 'specificity' and 'power' of the study, respectively. Incorrect results include Type I errors (concluding *D*_1 _when *H*_0 _is true), and Type II errors (concluding *D*_0 _when *H*_1 _is true); the corresponding probabilities *Pr*(*D*_1_*|H*_0_) and *Pr*(*D*_0_*|H*_1_) are called the 'Type I error rate' and 'Type II error rate', respectively. There is a perfect analogy (and mathematically, no difference) between these probabilities and the 'four fundamental forward probabilities' well known to physicians in the context of diagnostic testing, namely, the true and false positive rates, and true and false negative rates. Similarly, corresponding to the 'four fundamental inverse probabilities' of diagnostic testing, namely positive and negative predictive values and the false detection rate and false omission rate, there are exactly analogous quantities for the hypothesis testing scenario, that is *Pr*(*H*_0_|*D*_0_), *Pr*(*H*_1_*|D*_1_), *Pr*(*H*_0_*|D*_1_), and *Pr*(*H*_1_*|D*_0_). (See the Additional file 1 for a brief review of the fundamental forward and backward probabilities of diagnostic testing.) This analogy is summarized in Table 3 and has been expounded beautifully in a classic paper by Browner and Newman [63]. We will return to this analogy near the end of the paper.

**Table 3:** The analogy between diagnostic tests and clinical trials.

Diagnostic testing	Clinical trials
Absence of disease	Truth of null hypothesis
Presence of disease	Falsity of null hypothesis
Cutoff between positive and negative results	Significance level, α
Test result	*P*-value
Negative result	*P*-value > α
Positive result	*P*-value < α
Sensitivity	Power
False positive rate (1-specificity)	Significance level, α
Prior probability of disease	Prior probability of a difference between groups
Posterior probability of disease, given test result	Posterior probability of a difference between groups, given study results

### The null hypothesis significance testing procedure

Let us now consider the conventional statistical reasoning process followed in drawing conclusions about experiments. This reasoning is prescribed by a standardized statistical procedure, the 'null hypothesis significance testing procedure' (NHSTP), or simply 'significance testing', consisting of the following steps.

1. Specify mutually exclusive and jointly exhaustive hypotheses *H*_0 _and *H*_1_.

2. Design an experiment to obtain data *D*, and define a test statistic, that is a number or series of numbers that summarize the data, *T *= *T *(*D*) (for example the mean or variance).

3. Choose a minimum acceptable level of Type I error, called the 'significance level', denoted α

4. Do the experiment, yielding data *D*, and compute the test statistic, *T *= *T *(*D*).

5. Compute the *P*-value of the data from the test statistic, *P *= *P *(*T *(*D*)).

6. Compare the *P*-value to the chosen significance level. If *P *≤ *α*, conclude that *H*_1 _is true. If *P *>*α*, conclude that *H*_0 _is true.

In the customary statistical jargon, when *P *≤ *α*, we say that the experimental results are 'statistically significant', otherwise, they 'do not reach significance.' Also, note that the *P*-value itself is a statistic, that is a number computed from the data, so in effect we compute a test statistic *T = T *(*D*), from which we compute a second test statistic *P = P *(*T*(*D*)).

### *P-*values

We now review what *P*-values mean. The technical definition that we will use differs in important ways informal definitions more familiar to physicians, and the difference turns out to be consequential, as witnessed by the existence of a large critical literature dealing with practical and philosophical problems arising from definitions in common use [5,7,13-17,26,28,50,64-83]. As an overview to our own discussion of the conceptual issues at stake, we note that the literature critical of *P*-values can be roughly divided into two dominant themes [75]. First, there are problems of interpretation. For example, consider the commonly encountered informal definition of the *P*-value as the probability that the observed result could have been produced by chance alone

The probability that the observed result could have been produced by chance alone

This definition is vague, and tempts many users into confusing the probability of the hypothesis given the data with the probability of the data given the hypothesis [13,17], that is it is unclear whether this definition refers to a conditional probability with the hypothesis H0 before the conditioning line, *Pr*(*H*0*|·*), or after the conditioning line, *Pr*(·*|H*0), which have very different meanings. Another common complaint is that the conventional cutoff value for 'significance' of *P *< 0.05 is arbitrary. Finally, many have argued that real-world null hypotheses of 'no difference' are essentially never literally true, hence with enough data a null hypothesis can essentially always be rejected with an arbitrarily small *P*-value, casting doubt on the intrinsic meaningfulness of any isolated statement that *'P *<*x*'. *A *second entirely different class of *P*-value criticisms concerns problems of construction [7,26,28,75,83]. This critique maintains that *P*-values as commonly conceived are in fact conceptually incoherent and meaningless, rather than simply being subject to misinterpretation. The charges revolve around a more explicit yet still mathematically informal type of definition of the *P*-value such as

the probability that the data (that is the value of the summary statistic for the data), or more extreme results, could have occurred if the intended experiment was replicated many, many times, assuming the null hypothesis is true.

The potential morass created by this definition can be illustrated by imagining that an experimenter submits a set of data, consisting, say, of 23 data samples, to a statistical computer program, which automatically computes a *P*-value. According to the definition above, to produce the *P*-value, the computer must implicitly make several assumptions, often violated in actual practice, about the experimenter's intentions, such as the assumption that there was no intention to: collect more or less data based on an analysis of the initial results (the 'optional stopping problem'); replace any lost data by collecting additional data; run various conditions again; or compare the data with other data collected under different conditions [26,28,75]. Any of these alternative intentions would leave the actual data in hand unaltered, while implicitly altering the null hypothesis, either trivially by changing the number of data points that would be collected in repeated experiments, or by more profound alterations of the precise mathematical form of the probability distribution describing the null hypothesis. Consequently, the *P*-value apparently varies with the unstated intentions of the experimentalist, which in turns means that, short of making unjustified assumptions about those intentions, the *P*-value is mathematically ill defined.

In what follows, we will avoid the 'constructional' objections raised above by using a mathematically explicit definition for the *P*-value. Problems with interpretation will still remain, and the following section will focus in detail on what we believe are the most serious of the common modes of misinterpreting *P*-values. The generally accepted mathematical definition for the *P*-value is [84]:

the probability under the null hypothesis of obtaining the same or even less likely data than that which is actually observed, that is the probability of obtaining values of the test statistic that are equal to or more extreme than the value of the statistic actually computed from the data, assuming that the null hypothesis is true.

Note that this definition does not include any reference to the 'intentions' under which the data were collected. To avoid any possible confusion, we emphasize that this definition requires that the null hypothesis, *H*0, be fully specified. This means, for example, that the number of data samples *n*, constituting the data *D*, the chosen data summary statistic *T *(*D*), and more generally a mathematical formula for the probability distribution of values for the data summary statistic under the null hypothesis, *Pr*(*T *(*D*)|*H*0), be explicitly stated. In some cases, this specification is straightforward. For example, if the data is assumed to follow a normal distribution, then the null hypothesis can be fully specified by simply stating values for two parameters, the mean and standard deviation. In other cases the distribution can have a mathematically complicated form. Methods for specifying and computing complex null hypotheses are beyond the scope of this essay, but have been well worked out in a wide variety of practically important cases, and are in wide use in the field of statistics. The important point to grasp here is that once the null hypothesis *H*0, is specified, or more precisely, the relevant probability distribution *Pr*(*T *(*D*)|H0), then computing the *P*-value can in principle proceed in a straightforward, uncontroversial manner, according to its mathematical definition given above. As mentioned above, without specifying the null hypothesis distribution explicitly, the *P*-value is ill-defined, because any raw data are generally consistent with multiple different possible sample-generation processes, each which of may entail a different *P*-value [25,26].

We now turn to explaining our final, technical definition of the *P*-value. We will do this by exploring the definition from the vantage point of three different examples. The third example presents an additional, alternative definition of *P*-values which provides novel insights into the true meaning of *P*-values by viewing them from the medically familiar perspective of sensitivity and specificity considerations, in the context of ROC curves. This final definition will be mathematically equivalent, though not in an immediately obvious way, to the definition just given.

#### Angle 1. P-values as tail area(s)

**Figure 6:**
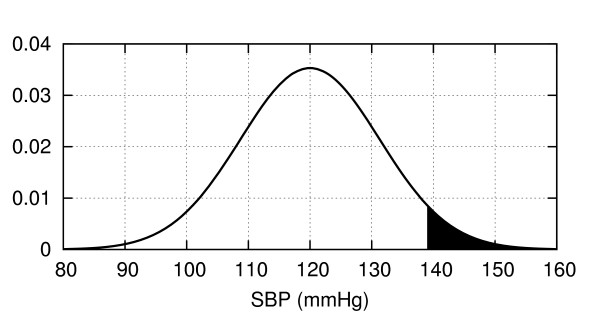
**Distribution of systolic blood pressures for a population of healthy 60-69 year old males (from data in **[125]**)**. The value *SBP *= 138.6 mmHg has a *P*-value of 0.05, equal to the shaded area under the curve.

Graphically, a *P*-value can be depicted as the area under one or two tails of the null-hypothesis probability distribution for the test statistic, depending on the details of the hypothesis being tested. For example, consider the classification of patients' systolic blood pressure as either chronically hypertensive, *H*_1_, or not chronically hypertensive, *H*_0_, on the basis of a single blood pressure measurement. Let us assume that blood pressures for normotensive patients obey a normal distribution N(BP), as shown in Figure 6. If for a particular patient we obtain a systolic blood pressure of *SBP *= 138.6, then the *P*-value for this result is the probability in a non-hypertensive patient of finding a blood pressure equal to or greater than this value, or the area under the right sided tail of N(BP), starting from *SBP *= *138.6*.

If instead the null hypothesis states that the patient is chronically normotensive, *H*_0_, so that the alternative *H*_1 _includes the possibility of either hypertension or hypotension, then the *P*-value would be 'two-sided', since values under an equally-sized left sided tail of the distribution would be equally contrary to the hypothesis *H*_0 _and hence would have caused us to reject *H*_0 _according to the null hypothesis significance testing procedure **(**NHSTP**)**.

#### Angle 2. P-values for coin flipping experiments

Let us carry out the *P*-value calculation in detail for a simple coin flipping experiment, where we wish to decide whether a coin is fair (equal probability of heads or tails) or biased (unequal probabilities). Note that the *P*-value in this case is 'two-sided'. Following the NHSTP:

1. Let *H*_0 _= 'the probability of heads is 1/2', *H*_1 _= 'probability of heads ≠ 1/2'.

2. The experiment will consist of flipping a coin a number of times *n*, and the data *D *will thus be a series of heads or tails. For our test statistic *T *, let us compute the difference between 1/2 and the fraction of heads, that is if *k *of the *n *coin tosses land as heads, then *T *(*D*) = |1/2-k/*n*|. For this example, let us put *n *= 10.

3. We set the significance level to the conventional value *α *= 0.05 = 5%.

4. Having done the experiment suppose we get data *D = *(*H, H, H, H, H, H, T, H, H, T*). This sequence contains eight heads, so *T *(*D*) =**|**1/2-8/10| = 0.3.

5. To calculate the *P*-value, we must consider all the ways in which the data could have been as extreme or more extreme than observed, assuming that the null hypothesis is true. That is, we need to consider all possible outcomes for the data *D *such that *T*(*D*) *≥ *0.3, and calculate the joint probability of these outcomes, assuming that the coin is fair. Clearly, observing eight, nine, or ten heads would be 'as extreme or more extreme' than our result of eight heads. Since the null hypothesis assumes equal probability for heads and tails, symmetry dictates that observing zero, one, or two heads would also qualify. Hence, the *P*-value is:

p=Pr(T(D)≥0.2|H0)=Pr(k≥8 or k≤2|H0)=10.94%.

(See Additional file 1 for details of this and the next two calculations.)

6. Since *p *≥ 5%, the NHSTP tells us to accept the null hypothesis, concluding that the coin is fair.

Before leaving this example, it is instructive to examine its associated Type I and II error rates. The Type I error rate (false positive rate) in this case is the probability of incorrectly declaring the coin unfair (*H*_1_) when in fact it is fair (*H*_0_), that is, the probability of getting *P *≤ *α *when in fact *H*_0 _is true. It turns out that had we observed just one more head then the NHSTP would have declared a positive result. That is, suppose *k *= 9, or *T *(*D*) = |1/2 - 9/10| = 0.4.

Then:

p=Pr(T(D)≥0.4|H0)=Pr(k≥9 or k≤1|H0)=2.15%.

Thus, we see that *P *≤ *α *whenever *d *≥ 0.4, hence the Type I error rate or false positive rate is:

$$FPR=Pr(D_{1}|H_{0})=Pr(p\leq \alpha |H_{0})=2.15\%.$$

Calculation of the false negative rate requires additional assumptions, because a coin can be biased in many (in fact, infinitely many) ways. Perhaps the least committed alternative hypothesis *H*_1 _is that for biased coins any heads probability different from 1/2 is equally likely. In this case the false negative rate turns out to be *FNR *= *Pr*(*D*_0_|*H*_1_) = 72.73%

#### Angle 3: P-values from ROC curves

To take a third angle, we consider an alternative definition for the *P*-value [84]. The *P*-value is

the minimum false positive rate (Type I error rate) at which the NHSTP will reject the null hypothesis.

Though not obvious at first glance, this definition is mathematically equivalent to our previous definition of the *P*-value as the probability of a result at least as extreme as the one we observe. The effort required to see why this is the case affords additional insight into the nature of *P*-values.

Let us step back and consider the null hypothesis testing procedure from an abstract point of view. The NHSTP is one instance of threshold-decision procedure, that is, a procedure that chooses between two alternatives by comparing a test statistic computed from the data *T*(*D*) with a threshold *γ *(in the case of the NHSTP, the statistic is the *P*-value, and the threshold is the significance level *α*). The procedure declares one result when the test statistic is less than or equal to threshold, and the alternative result when the threshold is exceeded. Identifying one of the alternatives as 'positive' and the other as 'negative', in general any such threshold-based decision procedure must have a certain false positive and false negative rate, determined by the chosen threshold. More explicitly, let us denote the positive and negative alternatives as *H*_1 _and *H*_0_, respectively, and declare a positive result whenever *T*(*D*) ≤ *γ*, or a negative result whenever *T*(*D*) >*γ*. A false positive then occurs if *T*(*D*) ≤ *γ *when in fact *H*_0 _is true, and the probability of this event is denoted *FPR*(*γ*) = *Pr*(*T*(*D*) ≤ *γ|H*_0_). Similarly, a true positive result occurs if *T*(*D*) ≤ *γ *when *H*_1 _is true, and the probability of this event is denoted *TPR*(*γ*) = *Pr*(*T*(*D*) ≤ *γ|H*_1_). If we allow the threshold to vary, we can generate a curve of the false positive rate versus the false negative rate; such a curve is called a ROC curve. To make this discussion concrete, let us return to our coin flipping example. In that case, we set the 'positive' alternative to *H*_1 _= 'the coin is biased' (that is *Pr*(*Heads|H*_1_) ≠1/2), and set the negative alternative to *H*_0 _= 'the coin is fair' (*Pr*(*Heads|H*_0_) = 1/2). Setting the test statistic as before to *T*(*D*) = *d *= |1/2 - *k*/*n*|, we then have:

$$\begin{array}{l} FPR(\gamma )=Pr(d\geq \gamma |H_{0}),\\ TPR(\gamma )=Pr(d\geq \gamma |H_{1}). \end{array}$$

**Figure 7:**
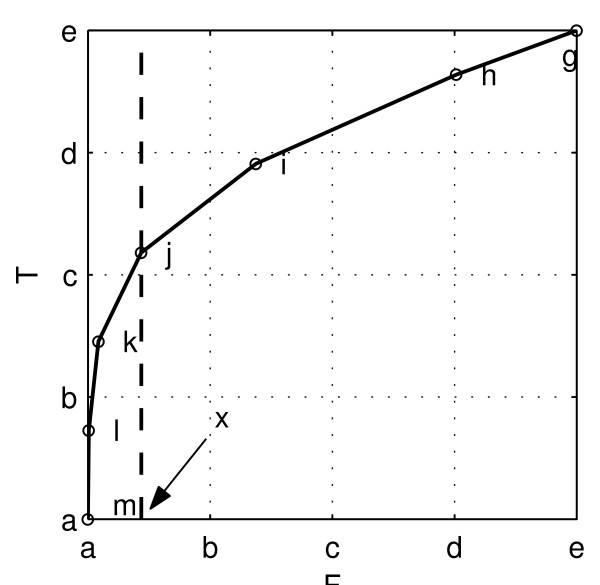
**ROC curve for the coin flipping experiment with *n *= 10, *H*_0 _: *Pr*(*Heads*) = 0.5 vs.H_1 _: *Pr*(*Heads*) = 0.7**. The curve is generated by varying a threshold between 0 (corresponding to the point (1, 1)) and 10 (corresponding to the point (0, 0)).

The resulting ROC curve *ROC*(*γ*) = (*FPR*(*γ*), *TPR*(*γ*)) is plotted in Figure 7. (On a technical note, the way we have set up our decision procedure, there are really only seven achievable values of (*TPR*(*γ*), *FPR*(*γ*)) on this ROC curve, marked by the circles: The first five values correspond to the five possible values of *d*, 0, 0.1, 0.2, 0.3, 0.4, which correspond in turn to the following pairs of possible values *k *for the number of heads in ten coin tosses (0. 10), (1. 9), (2. 8), (3. 7), (4. 6) (each member of the pair gives the same value for *d*); the sixth value corresponds to the value *d *= 0.5, which corresponds to a result of five heads; and the seventh value corresponds to setting the threshold to any value beyond what is obtainable, that is to *γ *< 0. We have connected these seven points with straight lines to create a more aesthetically pleasing plot.)

Key points on the ROC curve are marked by circles, and the corresponding value for is *γ *noted. Points on the ROC curve 'down and to the left' (low false positive rate, low true positive rate) correspond to setting the threshold low; whereas values 'up and to the right' (high false positive rate, high true positive rate) correspond to setting the threshold high. Clearly, if we wished to avoid all false positive conclusions, we could set the threshold to -∞, since all results will then be declared negative (*Pr*(*d *≤ -∞|*H*_0_) = 0), but this comes at the expense of rejecting all true positive results as well (since *Pr*(*d *≤ -∞|*H*_1_) = 0). Conversely, we can avoid missing any true positive results by setting the threshold to *γ *≤ 0.5, since it is true for all possible results that *d *≤ 0.5 (hence *P*r(*d *≤ 0.5|*H*_1_) = 1), but this simultaneously results in a maximal false positive rate (since *P*r(*d *≤ 0.5|*H*_0_) = 1 also). Clearly, positive results are only meaningful when obtained with the threshold *γ *set to some value intermediate between these extremes. Now, suppose that after conducting our coin flipping experiment we decide to 'cheat' as follows. As before let the outcome be that we get eight heads, or *d *= |1/2 - 8/10| = 0.3. Rather than choosing the decision threshold beforehand, we instead choose the threshold after seeing this result, to ensure that the result is declared positive. Our results will look best if we choose the threshold *γ *as small as we can, to let through as few false positives as possible, while still letting our result pass. This special choice of the threshold *γ *is clearly the value of our actual result, so we set *γ *= *d *= 0.3, and voilà, our result is positive. We cannot make the false positive rate any smaller without making our result negative according to the NHSTP.

Now for the point of this whole exercise: If we drop a vertical line from the point on the ROC curve *ROC*(0.3) = (*TPR*(0.3), *FPR*(0.3)) down to the x-axis to see where it intersects, we see that the false positive rate is *FPR*(0.3) = 10.94%, which is the result we calculated previously as the *P*-value. Thus the condition for declaring a positive result (*d *≤ *γ*) is equivalent to the condition in the NHSTP (*P *≤ *α*), hence, as claimed, the *P*-value is the minimum false positive rate at which the NHSTP will reject the null hypothesis. As an immediate corollary we also see that false positive rate of the NHSTP is simply the significance level, that is:

$$Pr(D_{1}|H_{0})=Pr(P\leq \alpha |H_{1})=\alpha$$

### Is significance testing rational?

The null hypothesis significance test (NHST) should not even exist, much less thrive as the dominant method for presenting statistical evidence. . . It is intellectually bankrupt and deeply flawed on logical and practical grounds. - Jeff Gill [85]

We are now in a position to answer the question: Is the null hypothesis significance testing procedure a rational method of inference? We will show momentarily that the answer is a resounding 'NO!', but first we briefly consider why, despite its faults, many find it intuitively plausible. Several books explore the reasons in detail [59,86-88], and a full account is well beyond the scope of this paper. We will focus on one particularly instructive explanation, called 'the illusion of probabilistic proof by contradiction' [13]. Consider once again the valid logical argument form:

$$\begin{array}{l} \text{if}\;A\;\text{is}\;\text{true,}\;\text{then}\;B\;\text{is}\;\text{true}\text{.}\\ B\;\text{is}\;\text{false}\text{.}\\ ∴A\;\text{is}\;\text{false}\text{.} \end{array}$$

This argument is called 'proof by contradiction': *A *is proved by 'contradicting' *B*, that is the falsehood of *A *follows from the fact that *B *is false. It is tempting to adapt this argument for use in uncertain circumstances, like so:

$$\begin{array}{l} \text{If}\;A\;\text{is}\;\text{true,}\;\text{then}\;B\;\text{is}\;\text{probably}\;\text{true}.\\ B\;\text{is}\;\text{false}.\\ ∴A\;\text{is}\;\text{false}\text{.} \end{array}$$

By analogy, this argument could be called 'probabilistic proof by contradiction'. However, this analogy quickly dissolves after a little reflection: The premise (that is the 'if, then' statement) leaves open the possibility that *A *may be true while *B *is nonetheless false. More concretely, consider the statement 'If a woman does not have breast cancer, then her mammogram will probably be negative.' (This example is discussed more extensively in an excellent online tutorial by Eliezer Yudkowsky [61].) This statement is true. However, given a positive mammogram, one cannot invariably pronounce a diagnosis of breast cancer, because false positives do sometimes occur. This simple example makes plain that 'probabilistic proof by contradiction' is an illusion - it is not a valid argument. And yet, this is literally the form of argument made by the NHSTP. To see this, simply make the following substitutions:

*A *= '*H*_0 _is true', and *B *= '*P *>*α*', to get:

$$\begin{array}{l} \text{If}\;H_{0}\;\text{is}\;\text{true,}\;\text{then}\;\text{probably}\;P\;>\;\alpha .\\ p\leq \alpha .\\ ∴H_{0}\;\text{is}\;\text{false}. \end{array}$$

Again, we have just seen that this is an invalid argument. One obvious 'fix' is to try softening the argument by making the conclusion probabilistic:

$$\begin{array}{l} \text{If}\;H_{0}\;\text{is}\;\text{true,}\;\text{then}\;\text{probably}\;P>\alpha .\\ P\leq \alpha .\\ ∴H_{0}\;\text{is}\;\text{probably}\;\text{false}. \end{array}$$

Unfortunately, any apparent validity this has is still an illusion. To see the problem with this argument, let us return to the mammography example. Is it rational to conclude that a positive mammogram implies that a woman probably has breast cancer? The correct answer, obvious to most physicians at an intuitive if not at a formal statistical level is, 'it depends on the patient's clinical characteristics, and on the quality of the test'. Very well, then let us give a bit more information: Suppose that mammography has a false positive rate of 20%, and sensitivity of 80%. Can we now assign a probable diagnosis of breast cancer? Interestingly, most physicians answer this question affirmatively, giving a probability of cancer of 80%, a conclusion apparently reached by erroneously replacing the sensitivity *Pr*(*H*_1_*|D*_1_) with the positive predictive value *Pr*(*D*_1_*|H*_1_) [9,11,12]. The fallacy here has been satirized thus:

It is like the experiment in which you ask a second-grader: 'If eighteen people get on a bus, and then seven more people get on the bus, how old is the bus driver?' Many second-graders will respond: 'Twenty-five.'....Similarly, to find the probability that a woman with a positive mammography has breast cancer, it makes no sense whatsoever to replace the original probability that the woman has cancer with the probability that a woman with breast cancer gets a positive mammography. - Eliezer Yudkowsky [61]

To calculate the desired probability *P*r(*H*_1_*|D*_1_) correctly, Bayes' rule requires that we also know the prior probability of disease. Suppose that our patient is a healthy young woman, from a population in which the prevalence of breast cancer is 1%. Then, given her positive mammogram the probability that she has breast cancer is:

$$Pr(H_{1}D_{1})=\frac{\left(.80)(.01\right)}{\left(.8)(.1)+(.99)(.2\right)}=7.8\%.$$

To put it as alarmingly as possible, the probability that she has breast cancer has increased by almost 8 fold! Nevertheless, she probably does not have cancer (7.8% is far short of 50%); the odds are better than nine to one against it, despite the positive mammogram. Thus, while further testing may be in order, a rational response is reassurance and perhaps further investigation rather than pronouncement of a cancer diagnosis. This and other examples familiar from everyday clinical experience make clear that the null hypothesis significance testing procedure cannot 'substitute' for Bayes' rule as a method of rational inference.

We have focused our criticism on what we consider to be the most fundamental and most common error in the interpretation of *P*-values, namely, the error of mistaking 'significant' *P*-values as proof that a hypothesis is 'probably true'. There are many other well documented conceptual problems with *P*-values as commonly employed which we have not discussed. The interested reader is referred to the excellent discussions in the following references [7,28].

### Answers to the quiz

The answer to the quiz at the beginning of this paper is plain from the preceding discussion. Given a *P*-value that reaches significance (such that the NHSTP would have us conclude that *H*_1 _is true), what conclusions are we actually justified in drawing regarding the probability that either hypothesis *H*_1 _or *H*_0 _is true? Answers (1), (2), and (5) are incorrect because the NHSTP, which corresponds to the 'hard' version of 'probabilistic proof by contradiction' is an invalid argument. Answers (3), (4), and (6) are invalid because the 'softened' version of the same argument is still invalid.

To determine the probability that *H*_1 _is actually true in light of the positive result *D*_1 _= '*P *<*α*', that is, to calculate *Pr*(*H*_1_*|D*_1_), Bayes' rule requires that we have three pieces of information. First, we need the false positive rate, which as we have seen for the NHSTP is *Pr*(*D*_1_*|H*_0_) = *Pr*(*P ≤ α|H*_0_) = *α*; this is the only piece of information we were given in the quiz question. Second and third, however, we need to know the 'power' (sensitivity) of the study, *Pr*(*D*_1_|*H*_1_), and the pre-test probability of the hypothesis, *Pr*(*H*_1_). Thus, the correct answer is '(7) None of the above'.

### Do prior probabilities exist in science?

Though most physicians are comfortable with the concept of prior probability in the context of diagnostic test interpretation, many are less comfortable thinking about prior probabilities in the context of interpreting medical research data. As one respondent to our quiz thoughtfully objected,

The big difference between a study and a clinical test is that there is no real way of knowing how likely or unlikely a hypothesis is a priori. In order to have a predictive value in a clinical test, you need a prevalence or pre-test probability. This does not exist in science. It is the job of the scientist to convince us that the pre-test probability is reasonably high so that a result will be accepted. They do this by laying the scientific groundwork (introduction), laying out careful methods, particularly avoiding bias and confounders (methods), and describing the results carefully. Thereafter, they use the discussion section to outright and unabashedly try to convince us their results are right. But in the end, we do the positive predictive value calculation in our head as we read a paper... As an example, one person reads the SPARCL study and says, 'I do not CARE that the *P*-value shows statistical significance, it is hooey to say that statins cause intracranial hemorrhage.'... They have set a very low pre-test probability in their head. Another person reads the same study and says, 'I have wondered about this because I have seen lots of bleeds in people on statins.' They have set a much higher pre-test probability.

This response actually makes our point, perhaps inadvertently, about the necessity of prior probabilities. Nevertheless, several important points raised by this response warrant comment.

#### Do prior probabilities 'exist' in science?

First, to the philosophical question of whether prior probabilities 'exist' in science, the answer is 'yes and no'. On the one hand, probability theory is always used as a simplifying model rather than a literal description of reality, whether in science or clinical testing (with the possible exception of probabilities in quantum mechanics). Thus, when one speaks of the probability that a coin flip will result in heads, that a drug will have the intended effect, or that a scientific theory is correct, one is not necessarily committing to the view that nature is truly random. In these cases, the underlying reality may be deterministic (for example a theory is either true or false), in which referring to probabilities represents merely a convenient simplification, but do not really 'exist' in the sense that they would not be needed in a detailed, fundamental description of reality. However, simplification is essentially always necessary in dealing with any sufficiently complex phenomena. For example, while it might be possible to conceive of a supercomputer capable of predicting the effects of a drug using detailed modeling of the molecular interactions between the drug and the astronomical number of cells and molecules in an individual patient's body, in practice we must make predictions with much less complete information, hence we use probabilities. The use of such simplifications is no less important in scientific thinking than in medical diagnostic testing. Thus, insofar as probabilities 'exist' at all, they are not limited to the arena of diagnostic testing.

#### Are prior probabilities in science arbitrary?

Given that prior probabilities for hypotheses in science and medicine are often difficult to specify explicitly in precise numerical terms, does this mean that any prior probability for a hypothesis is as good as any other? There are at least two reasons that this is not the case. First, pragmatically, people do not treat prior probabilities regarding scientific or medical hypotheses as arbitrary. To the contrary, they go to great lengths to bring their probabilities into line with existing evidence, usually by integrating multiple information sources, including direct empirical experience, relevant theory (for example an understanding of physiology), and literature concerning prior work on the hypothesis or related hypotheses. These prior probability assignments help scientists and physicians choose which hypotheses deserve further investment of time and resources. Moreover, while these probability estimates are individualized, this does not imply that each person's 'subjective' estimate is equally valid. Generally, experts with greater knowledge and judgement can be expected to arrive at more intelligent prior probability assignments, that is their assignments can be expected to more closely approximate the probability an 'ideal observer' would arrive at based on optimally processing all of the existing evidence. Second, in a more technical vein, methods for estimating accurate prior probabilities from existing data are an active topic of research, and are likely to lead to increased and more explicit use of 'Bayesian statistics' in the medical literature [29,31-36,83,89].

#### Taking responsibility for prior probabilities

Finally, regarding the responsibility of scientific authors and readers to take prior probabilities seriously: We emphatically agree that authors should strive to place their results in context, so as to give the firmest idea possible of how much plausibility one should afford a hypothesis, prior to seeing the new data being presented. Without this context, there is no way to appraise how likely a hypothesis is to actually be true, or how strong the evidence needs to be to be truly persuasive. The neglect of thorough introductory and discussion sections in scientific papers is decried by many as a natural side effect of reliance on significance testing arguments [7,90,91], and is blamed for the too-common phenomenon of unreproducible results in clinical trials [92-97], and has even lead some authors to suggest that the majority of published medical research results may be false [5,98-100]. Similarly, it is a central thesis of this paper that in reading the medical literature physicians should strive to take prior probabilities into account. Indeed, taking prior probabilities into account can be viewed as a good summary of what it means to read the medical literature critically.

### Has significance testing been perverted?

Considering the criticisms we have reviewed, it is natural to ask whether significance testing is being used as its originators intended. Significance testing is actually an amalgam of two approaches to statistical inference, developed on the one hand by RA Fisher, who invented the concept of *P*-values, and on the other hand by J Neyman and K Pearson, who together developed the theory of binary hypothesis testing. Hypothesis testing and *P*-values were combined into the method of null hypothesis significance testing by others, to the chagrin of Fisher, Neyman and Pearson, who were vigorously outspoken critics of one another's methods [16,17]. In this connection, the following quotation from Neyman and Pearson on their philosophy towards hypothesis testing (of which significance testing is a special case) is particularly interesting:

...no test based upon a theory of probability can by itself provide any valuable evidence of the truth or falsehood of a hypothesis. . . But we may look at the purpose of tests from another viewpoint. Without hoping to know whether each separate hypothesis is true or false, we may search for rules to govern our behavior with regard to them, in following which we insure that, in the long run of experience, we shall not often be wrong [101].

Thus, Neyman and Pearson apparently did not intend hypothesis testing to be used as it usually is used nowadays, as a method for appraising the truth of individual hypotheses. Rather, their method was intended merely to be correct in an aggregate sense. While this may be acceptable, say, to decide the fates of mass-produced objects in an industrial setting, it is unsatisfactory in medical situations involving individuals. There, it is imperative that we strive to be right in each case. Similarly, few researchers would be content to use a method of inference realizing that it cannot accurately appraise the truth of the individual hypotheses. While significance testing does not provide a way to know 'whether each separate hypothesis is true or false', fortunately Bayes' rule does provide rational grounds for appraising the strength of evidence in favor of individual hypotheses.

### How significant is a significant result?

**Figure 8:**
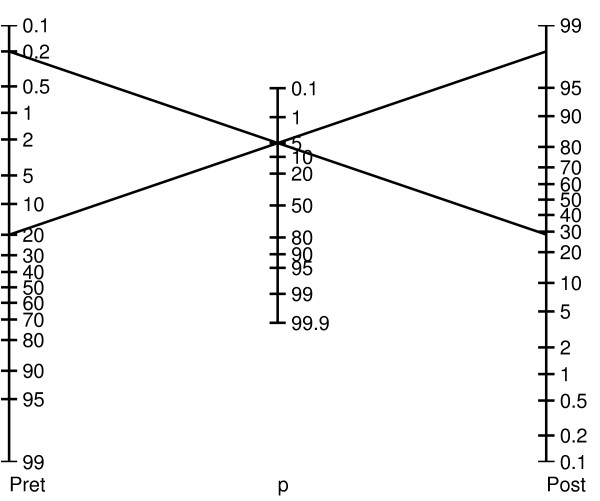
**Bayesian *P*-value nomogram for a hypothetical hypothesis testing problem**. This nomogram is calculated assuming normal distributions for the null hypothesis *H*_0 _and the alternative hypothesis *H*_1_, with variance equal to one, and means differing by a value of two.

If it is unjustified to regard a 'statistically significant' result as sufficient evidence for the truth of a hypothesis, then what can we conclude when we read '*P *≤ *α*'? How much evidence does a statistically significant result provide for its hypothesis? The fact is that the amount of evidence provided by a *P*-value depends on the prior probability and power of the research methodology, in the way prescribed by Bayes' rule. Thus, there is no generic value of *P *that will render a hypothesis more likely true than not (that is *Pr*(*H*_1_*|P *≤ *α*) > 50%). Rather, the true 'significance' of *P *varies from case to case, in the same way as the meaning of a BNP value varies according to a patient's clinical characteristics when evaluating for suspected congestive heart failure (see Additional file 1 Figure S1) [102,103]. It is helpful conceptually when assessing *P*-values to envision a *'P*-value nomogram', as illustrated in Figure 8. As shown, a *P*-value of 0.05 can lead to very different posterior probabilities. Note that the particular nomogram shown is not universal; it was calculated by assuming specific distributions for *H*_0 _and *H*_1_. But the basic idea that the degree of support for a hypothesis provided by a *P*-value depends on the pre-test probability is general.

### Are physicians good Bayesians?

Probability theory was regarded by its early architects as a model not only for how educated minds should work, but for how they do actually work. This 'probabilistic theory of mind' forms the basis for modern views on the nature of rationality in philosophy, economics, and more recently in neuroscience [104-108]. How can this be, when there is widespread misunderstanding of the most basic of statistical concepts like *P*-values and significance testing, even among a group as educated and accustomed to consuming statistical data as physicians? We briefly consider arguments for and against the possibility that physicians are, or can be, good Bayesians.

### Anti-Bayes

In his evaluation of the evidence, man is apparently not a conservative Bayesian: he is not a Bayesian at all. - Kahneman and Tversky [109]

The most serious challenge to the probabilistic theory of mind is the 'heuristics and biases' movement of experimental psychology, started by a series of influential papers published in the late 1960 s and early 1970 s by Kahneman and Tversky [109,110]. The central claim of this movement is that people tend to make judgements under uncertainty not according to Bayes' rule, but instead by simplifying rules of thumb (heuristics) that, while convenient, nevertheless often lead to systematic errors (biases). With respect to medical reasoning, we can roughly categorize the types of biases by whether they affect one's clinical estimates of prior or posterior probabilities.

#### Prior (pre-test) probabilities

Physicians' estimates of the prior probability of disease may vary wildly [10,111,112]. For example, given the same vignette of the history, physical exam, and EKG for 58 year old female with chest pain, physicians were asked to assign probabilities to various diagnoses including acute myocardial infarction (AMI), aortic dissection, and gastroesophageal reflux. Estimates for AMI ranged from 1% to 99%, and the probabilities assigned by many physicians surveyed added to greater than 100% [10]. Two classic examples of cognitive biases that contribute to this variability are the representativeness and availability biases.

##### Representativeness bias

This is the tendency to violate the old medical maxim, 'when you hear hoofbeats, think horses, not zebras.' That is, the tendency to set the prior probability inappropriately high for rare diseases whose typical clinical presentation matches the case at hand, and inappropriately low for common diseases for which the presentation is atypical. This bias leads to overdiagnosis of rare diseases.

##### Availability bias

Also called the 'last case bias' in the medical context, this is the tendency to overestimate the probability of diagnoses that easily come to mind, as when, having recently seen a case of Hashimoto's encephalopathy, one automatically suspects this first in the next patient who presents with confusion, a relatively nonspecific sign. Another example is doubting that smoking is harmful because one's grandmother was a smoker yet lived to age ninety.

#### Posterior (post-test) probabilities

Other studies have explored ways in which physicians deviate from Bayes' rule in updating prior probabilities in light of new data [113,114]. Well known examples of responsible underlying cognitive biases are the anchoring, confirmation, and premature closure biases.

##### Anchoring bias

This is the tendency to set one's posterior probability estimate inappropriately close to a starting value, called an anchor. Errors can arise from anchoring to an irrelevant piece of information (as when patients are sent home from the low-acuity part of the emergency department who would have been admitted from the high-acuity part), or by generally undervaluing new information when it does not support one's initial impression.

##### Confirmation bias

Also known as belief preservation, hypothesis locking, and selective thinking, this is the tendency maintain one's favored hypothesis by overvaluing and selectively searching for confirmatory evidence and undervaluing or ignoring contradictory evidence. Reasons for this bias include vested emotional interest, for example as when avoiding a potentially upsetting diagnosis, or inconvenience, for example as when downplaying medical symptoms in a patient with challenging psychiatric problems.

##### Premature closure bias

This is the tendency to make a diagnosis before sufficient evidence is available. Premature closure bias can arise from emotional factors such as discomfort over a patient's or the physician's own uncertainty, or because of time pressure [113,114].

### Pro-Bayes

[T]he theory of probability is at bottom nothing more than good sense reduced to a calculus which evaluates that which good minds know by a sort of instinct, without being able to explain how with precision. - Laplace [115]

The heuristics and biases movement notwithstanding, the probabilistic theory of cognition has been resurrected in recent years in the fields of neuroscience, artificial intelligence, and human cognitive science. As mentioned earlier, Bayesian theories have provided successful explanations of the sub- or pre-conscious mental phenomena, such as learning [40], visual object and pattern recognition [45,116], language learning and speech recognition [38,41]; and memory [42]. In the artificial intelligence community, there is a general consensus that many difficult engineering problems are best formulated and solved within a probabilistic framework, including computer vision, speech recognition, search engine technology, and pattern recognition, [43,44,46-48,50,51,53]. Similarly, Bayesian inference has become the generally accepted framework for understanding how the nervous system achieves its feats, yet unmatched by engineering technology, of visual and auditory perception, among other tasks [104,108,117-119]. The thread tying these various problems and fields together is the need to draw rich inferences from sparse data, that is, to reason under uncertain conditions where the required conclusions are underdetermined by the available evidence.

There is also a growing consensus that many higher-level human cognitive processes also operate on Bayesian principles [20,39,40]. Specific examples include studies of human symbolic reasoning [120], reasoning about and predicting the actions of other people [121], and estimating various everyday quantities [122]. Taking this last example as a case in point, Tenenbaum *et al*. recently studied the abilities of subjects to predict the values of uncertain quantities that arise in everyday reasoning situations. Subjects were told how long a particular everyday process had been going on so far (for example how long a cake had been baking, or how long a man had lived so far), and were asked to predict the final value of the process (for example how much longer before the cake will be done baking, or when the man will die). The scenarios tested included total final profits for movies, total runtimes of movies, the length's of poems, term lengths for US representatives, and cake baking times. In these tasks, people's judgements are remarkably close to optimal Bayesian estimates. These findings suggest that in many everyday tasks at which people are 'experts', people implicitly use the appropriate statistical distributions and, albeit unawares, carry out optimal probabilistic calculations.

### Instinctual Bayesianism?

How can the view that in many situations people perform Bayesian inference be reconciled with findings from the Heuristics and Biases movement (and our quiz results), showing that most people understand the elementary concepts of probability and statistics poorly at best? In large part, the answer is that fluency with statistics and probability theory at a formal level need not cast doubt on Laplace's claim that 'good minds' use probability theory by 'a sort of instinct'. Thus, although physicians are vulnerable to the traps of experimental psychologists in tests of formal verbal reasoning about probability and statistics, nevertheless physicians are adept at managing uncertainty. We suspect that studies similar to that of Tenenbaum *et al*. will ultimately show that, when dealing with uncertain situations they encounter often, good physicians frequently are much better Bayesians than the Heuristics and Biases movement gives them credit for.

## Summary

Until recently, the art of medical reasoning has arguably gotten along well enough with little formal understanding of mathematical probability. This has been possible largely because, as Laplace observed, at some informal, implicit level, the everyday reasoning of good minds conforms to the laws of probability. However, physicians can no longer afford the luxury of complete informality. Without a solid understanding of basic probability, one can no longer intelligently interpret the medical literature. The quiz results that began this essay are a sobering reminder that most physicians still lack understanding of elementary probability and statistics. In particular, it is worrisome that physicians seem to so easily fall prey to the illusion that significance testing allows one to evaluate the truth of a hypothesis without having to take into account contextual information like prior studies and biological plausibility.

Like others we are concerned that the increasing use of statistics without a parallel increase in statistical literacy renders the medical literature at risk for becoming less scientific [7,90,91,123,124]. Nevertheless, all statistical argumentation ultimately boils down to the basic question answered by Bayes' rule: In what way should one's confidence in a particular claim change in response to new data? Thus, a deeper appreciation of Bayes' rule may go a long way toward making physicians less vulnerable to the fallacies inherent in conventional applications of statistical significance testing.

## Abbreviations

AMI: acute myocardial infarction; NHST: null hypothesis significance test; NHSTP: null hypothesis significance testing procedure.

## Competing interests

The authors declare that they have no competing interests.

## Authors' contributions

A zero difference between the three authors' contributions to this work is among the credible values.

## Pre-publication history

The pre-publication history for this paper can be accessed here:

http://www.biomedcentral.com/1741-7015/9/20/prepub

## Supplementary Material

Additional file 1 **Supplemental material**. Click here for file

## References

[B1] Olser W Silverman M Murray T Bryan C The Quotable Osler 2003 Philadelphia: ACP Press

[B2] Horton NJ Switzer SS Statistical methods in the journal The New England Journal of Medicine 2005 353 18 1977 1979 10.1056/NEJM200511033531823 16267336

[B3] Altman DG Bland JM Improving doctors' understanding of statistics Journal of the Royal Statistical Society. Series A (Statistics in Society) 1991 154 2 223 267 10.2307/2983040

[B4] Windish DM Huot SJ Green ML Medicine residents' understanding of the biostatistics and results in the medical literature The Journal of the American Medical Association 2007 298 9 1010 1022 10.1001/jama.298.9.1010 17785646

[B5] Ioannidis JPA Why most published research findings are false PLoS Medicine 2005 2 8 e124 10.1371/journal.pmed.0020124 16060722 PMC1182327

[B6] Friedman SB Phillips S What's the difference? Pediatric residents and their inaccurate concepts regarding statistics Pediatrics 1981 68 5 644 646 7312466

[B7] Goodman SN Toward evidence-based medical statistics. 1: the P value fallacy Annals of Internal Medicine 1999 130 12 995 1004 10383371 10.7326/0003-4819-130-12-199906150-00008

[B8] Ghosh A Ghosh K Erwin P Do medical students and physicians understand probability? Quarterly Journal of Medicine 2004 97 53 55 http://qjmed.oxfordjournals.org 10.1093/qjmed/hch010 14702512

[B9] Casscells W Schoenberger A Graboys TB Interpretation by physicians of clinical laboratory results The New England Journal of Medicine 1978 299 18 999 1001 10.1056/NEJM197811022991808 692627

[B10] Cahan A Gilon D Manor O Paltiel O Probabilistic reasoning and clinical decision-making: do doctors overestimate diagnostic probabilities? Quarterly Journal of Medicine 2003 96 10 763 769 http://qjmed.oxfordjournals.org/cgi/content/abstract/96/10/763 10.1093/qjmed/hcg122 14500863

[B11] Eddy DM Probabilistic Reasoning in Clinical Medicine: Problems and Opportunities 1982 Cambridge, UK: Cambridge University Press 249 267

[B12] Gigerenzer G Hoffrage U How to improve Bayesian reasoning without instruction: frequency formats Psychological Review 1995 102 4 684 704 http://www.google.com/url?sa=t&source=web&cd=2&ved=0CBkQFjAB&url=http%3A%2F%2Fciteseerx.ist.psu.edu%2Fviewdoc%2Fdownload%3Bjsessionid%3D32E0F35DFDDC7AA54764A877EBC6825A%3Fdoi%3D10.1.1.128.3201%26rep%3Drep1%26type%3Dpdf&rct=j&q=How%20to%20improve%20Bayesian%20Reasoning%20Without%20Instruction%3A%20Frequency%20formats%20no%20notes%20yet%20&ei=bJJ3TYnpEcGY8QPAyoCgDA&usg=AFQjCNHsbqpkhuNll0H7hfT4pQ6wXI9QMg&cad=rja 10.1037/0033-295X.102.4.684

[B13] Falk R Greenbaum CW Significance tests die hard: the amazing persistence of a probabilistic misconception Theory Psychology 1995 5 75 98 10.1177/0959354395051004

[B14] Dienes Z Understanding Psychology as a Science: An Introduction to Scientific and Statistical Inference 2008 Basingstoke: Palgrave Macmillan http://www.loc.gov/catdir/enhancements/fy0828/2008014353-t.html

[B15] Gill J The insignificance of null hypothesis significance testing Political Research Quarterly 1999 52 3 647 674

[B16] Gigerenzer G Murray DJ Cognition as Intuitive Statistics 1987 Hillsdale, NJ: L. Erlbaum Associates

[B17] Gigerenzer G Keren G, Lewis C The superego, the ego, and the id in statistical reasoning A Handbook for Data Analysis in the Behavioral Sciences: Methodological Issues 1993 Hillsdale, NJ: L. Erlbaum Associates 574

[B18] Campbell L Garnett W The Life of James Clerk Maxwell. With a Selection from His Correspondence and Occasional Writings and a Sketch of His Contributions to Science 1882 London: Macmillan and Co

[B19] Mumford D The dawning of the age of stochasticity Mathematics: Frontiers and Perspectives 2000 197 218

[B20] Oaksford M Chater N Bayesian Rationality: The Probabilistic Approach to Human Reasoning 2007 Oxford: Oxford University Press [*Oxford Cognitive Science Series*] 10.1017/S0140525X09000284 19210833

[B21] Jaynes ET Bretthorst GL Probability Theory: The Logic of Science 2003 Cambridge, UK: Cambridge University Press

[B22] Cox RT The Algebra of Probable Inference 1961 Baltimore: Johns Hopkins Press

[B23] Jaynes ET Erickson GJ, Smith CR How does the brain do plausible reasoning? Maximum-Entropy and Bayesian Methods in Science and Engineering 1988 Kluwer Academic Publishers

[B24] Horn KSV Constructing a logic of plausible inference: a guide to Cox's theorem International Journal of Approximate Reasoning 2003 34 3 24 10.1016/S0888-613X(03)00051-3

[B25] Kruschke JK Doing Bayesian Data Analysis: A Tutorial with R and BUGS 2010 Academic Press

[B26] Kruschke JK Bayesian data analysis Wiley Interdisciplinary Reviews: Cognitive Science 2010 1 5 658 676 10.1002/wcs.72 26271651

[B27] Fisher RA Statistical Methods and Scientific Inference 1973 3, rev. and enl New York: Hafner Press

[B28] Kruschke JK What to believe: Bayesian methods for data analysis Trends in Cognitive Sciences 2010 14 7 293 300 10.1016/j.tics.2010.05.001 20542462

[B29] Gelman A Bayesian Data Analysis 2004 2 Boca Raton, Fla: Chapman & Hall/CRC [*Texts in Statistical Science*]

[B30] Diamond GA Kaul S Prior convictions: Bayesian approaches to the analysis and interpretation of clinical megatrials Journal of the American College of Cardiology 2004 43 11 1929 1939 10.1016/j.jacc.2004.01.035 15172393

[B31] Berry DA Bayesian clinical trials Nature Reviews. Drug Discovery 2006 5 27 36 10.1038/nrd1927 16485344

[B32] Goodman SN Introduction to Bayesian methods I: measuring the strength of evidence Clinical Trials (London, England) 2005 2 4 282 290 discussion 301-304, 364-378 16281426 10.1191/1740774505cn098oa

[B33] Berry DA Introduction to Bayesian methods III: use and interpretation of Bayesian tools in design and analysis Clinical Trials (London, England) 2005 2 4 295 300 discussion 301-304, 364-378 16281428 10.1191/1740774505cn100oa

[B34] Louis TA Introduction to Bayesian methods II: fundamental concepts Clinical Trials (London, England) 2005 2 4 291 294 discussion 301-304, 364-378 16281427 10.1191/1740774505cn099oa

[B35] Spiegelhalter DJ Myles JP Jones DR Abrams KR Bayesian methods in health technology assessment: a review Health Technology Assessment (Winchester, England) 2000 4 38 1 130 11134920

[B36] Spiegelhalter DJ Myles JP Jones DR Abrams KR Methods in health service research. An introduction to Bayesian methods in health technology assessment British Medical Journal (Clinical Research Ed.) 1999 319 7208 508 512 10454409 10.1136/bmj.319.7208.508 PMC1116393

[B37] Jacobs RA Kruschke JK Bayesian learning theory applied to human cognition Wiley Interdisciplinary Reviews: Cognitive Science 2010 2 8 21 10.1002/wcs.80 26301909

[B38] Chater N Manning CD Probabilistic models of language processing and acquisition Trends in Cognitive Sciences 2006 10 7 335 344 10.1016/j.tics.2006.05.006 16784883

[B39] Chater N Oaksford M The Probabilistic Mind: Prospects for Bayesian Cognitive Science 2008 Oxford: Oxford University Press

[B40] Tenenbaum JB Griffiths TL Kemp C Theory-based Bayesian models of inductive learning and reasoning Trends in Cognitive Sciences 2006 10 7 309 318 10.1016/j.tics.2006.05.009 16797219

[B41] Xu F Tenenbaum JB Word learning as Bayesian inference Psychological Review (New York) 2007 114 2 245 10.1037/0033-295X.114.2.245 17500627

[B42] Steyvers M Griffiths TL Dennis S Probabilistic inference in human semantic memory Trends in Cognitive Sciences 2006 10 7 327 334 10.1016/j.tics.2006.05.005 16793324

[B43] Manning CD Schütze H Foundations of Statistical Natural Language Processing 1999 MIT Press

[B44] Westover M O'Sullivan J Achievable rates for pattern recognition Information Theory, IEEE Transactions on 2008 54 299 320 10.1109/TIT.2007.911296 PMC7062371 32153303

[B45] Yuille A Kersten D Vision as Bayesian inference: analysis by synthesis? Trends in Cognitive Sciences 2006 10 7 301 308 10.1016/j.tics.2006.05.002 16784882

[B46] Grenander U Miller M Pattern Theory: From Representation to Inference 2007 USA: Oxford University Press

[B47] Jordan MI (Ed) Learning in Graphical Models 1998 1 The MIT Press

[B48] Bishop CM Pattern Recognition and Machine Learning 2007 1 Springer

[B49] Mumford D Desolneux A Pattern Theory: The Stochastic Analysis of Real-World Signals (Applying Mathematics) 2010 Natick, Mass.: A K Peters

[B50] MacKay DJC Information Theory, Inference & Learning Algorithms 2002 1 Cambridge University Press

[B51] Frey BJ Graphical Models for Machine Learning and Digital Communication 1998 The MIT Press

[B52] Jelinek F Statistical Methods for Speech Recognition 1998 The MIT Press

[B53] Pearl J Probabilistic Reasoning in Intelligent Systems: Networks of Plausible Inference 1998 Rev. 2nd printing San Francisco, Calif: Morgan Kaufmann [*The Morgan Kaufmann Series in Representation and Reasoning*]

[B54] Paté-Cornell E Guikema S Probabilistic modeling of terrorist threats: a systems analysis approach to setting priorities among countermeasures Military Operations Research 2002 7 4 5 20

[B55] Forrester M Pettitt A Gibson G Bayesian inference of hospital-acquired infectious diseases and control measures given imperfect surveillance data Biostatistics 2007 8 2 383 10.1093/biostatistics/kxl017 16926230

[B56] de Campos L Fernández-Luna J Huete J Bayesian networks and information retrieval: an introduction to the special issue Information Processing & Management 2004 40 5 727 733

[B57] Kolmogorov AN Foundations of the Theory of Probability 1956 2d english New York: Chelsea Pub. Co

[B58] Bayes T An essay towards solving a problem in the doctrine of chances Philosophical Transactions of the Royal Society 1763 53 370 418 10.1098/rstl.1763.0053

[B59] Gigerenzer G Swijtink Z Porter T Daston L Beatty J Kruger L The Empire of Chance: How Probability Changed Science and Everyday Life (Ideas in Context) 1989 Cambridge [Cambridgeshire]: Cambridge University Press

[B60] Krüger L The Probabilistic Revolution 1987 Cambridge, Mass: MIT Press

[B61] Yudkowsky E An Intuitive Explanation of Bayes' Theorem 2010 http://yudkowsky.net/rational/bayes

[B62] Dienes Z Book Website for: Understanding Psychology as a Science: An Introduction to Scientific and Statistical Inference, Palgrave Macmillan 2010 http://www.lifesci.sussex.ac.uk/home/Zoltan_Dienes/inference

[B63] Browner WS Newman TB Are all significant P values created equal? The analogy between diagnostic tests and clinical research The Journal of the American Medical Association 1987 257 18 2459 2463 10.1001/jama.257.18.2459 3573245

[B64] Cohen J The earth is round (p < .05) American Psychologist 1994 49 12 997 1003 http://web.math.umt.edu/wilson/Math444/Handouts/Cohen94_earth_is_round.pdf 10.1037/0003-066X.49.12.997

[B65] Cortina JM Dunlap WP On the logic and purpose of significance testing Psychological Methods 1997 2 2 161 172 http://www.sciencedirect.com/science/article/B6WYR-46P4P35-8/2/ac50f4ba827ae4cd48cd72896441afbc 10.1037/1082-989X.2.2.161

[B66] Dixon P The p-value fallacy and how to avoid it Canadian Journal of Experimental Psychology = Revue Canadienne De Psychologie Exp'erimentale 2003 57 3 189 202 14596477 10.1037/h0087425

[B67] Frick RW The appropriate use of null hypothesis testing Psychological Methods 1996 1 4 379 390 10.1037/1082-989X.1.4.379

[B68] Hagen RL In praise of the null hypothesis statistical test American Psychologist 1997 52 15 24 10.1037/0003-066X.52.1.15

[B69] Killeen PR An alternative to null-hypothesis significance tests Psychological Science: A Journal of the American Psychological Society/APS 2005 16 5 345 353 [PMID: 15869691] 15869691 10.1111/j.0956-7976.2005.01538.x PMC1473027

[B70] Killeen ACO jan Wagenmakers E Grünwald P A Bayesian perspective on hypothesis testing Psychological Science 2006 17 10.1111/j.1467-9280.2006.01758.x 16866752

[B71] Loftus GR Psychology will be a much better science when we change the way we analyze data Current Directions in Psychological Science 1996 5 6 161 171 [ArticleType: research-article/Full publication date: Dec., 1996/Copyright © 1996 Association for Psychological Science] 10.1111/1467-8721.ep11512376

[B72] Loftus GR Pashler H, Hoboken NJ Analysis, interpretation, and visual presentation of experimental data Stevens' Handbook of Experimental Psychology 2002 USA: John Wiley & Sons, Inc http://onlinelibrary.wiley.com/doi/10.1002/0471214426.pas0409/full

[B73] Nickerson RS Null hypothesis significance testing: a review of an old and continuing controversy Psychological Methods 2000 5 2 241 301 [PMID: 10937333] 10.1037/1082-989X.5.2.241 10937333

[B74] Trafimow D Hypothesis testing and theory evaluation at the boundaries: surprising insights from Bayes's theorem Psychological Review 2003 110 3 526 535 [PMID: 12885113] 10.1037/0033-295X.110.3.526 12885113

[B75] Wagenmakers EJ A practical solution to the pervasive problem of p values Psychonomic Bulletin & Review 2007 14 5 779 804 10.3758/bf03194105 18087943

[B76] Wainer H One cheer for null hypothesis significance testing Psychological Methods 1999 4 2 212 213 10.1037/1082-989X.4.2.212

[B77] Berger JO Wolpert RL The Likelihood Principle 1988 IMS

[B78] O'Hagan A Forster J Kendall's advanced theory of statistics. Vol. 2B: Bayesian inference 2004 http://adsabs.harvard.edu/abs/2004kats.book.....O

[B79] Royall R Statistical Evidence: A Likelihood Paradigm 1997 Chapman & Hall/CRC

[B80] Sellke T Bayarri M Berger J Calibration of *ρ *values for testing precise null hypotheses The American Statistician 2001 55 62 71 10.1198/000313001300339950

[B81] Stuart A Ord J Arnold S Kendall's advanced theory of statistics. Vol. 2a: classical inference and the linear model 1999

[B82] Goodman SN p values, hypothesis tests, and likelihood: implications for epidemiology of a neglected historical debate American Journal of Epidemiology 1993 137 5 485 496 discussion 497-501 8465801 10.1093/oxfordjournals.aje.a116700

[B83] Goodman SN Toward evidence-based medical statistics. 2: the Bayes factor Annals of Internal Medicine 1999 130 12 1005 1013 10383350 10.7326/0003-4819-130-12-199906150-00019

[B84] Wasserman L All of Statistics: A Concise Course in Statistical Inference 2004 New York: Springer [*Springer Texts in Statistics*]

[B85] Gill J 'S'attaquer a l'Heritage de Fisher: Comment Tester une Hypothese en Science Sociale: Quelques Commentaires Sur Denis.' (Grappling with Fisher's legacy in social science hypothesis testing: some comments on Denis.) Journal de la Société Franc¸aise de Statistique 2004 145 1 9

[B86] Matthews JR Quantification and the Quest for Medical Certainty 1995 Princeton, NJ: Princeton University Press

[B87] Marks HM The Progress of Experiment: Science and Therapeutic Reform in the United States, 1900-1990 2000 1st pbk Cambridge, UK: Cambridge University Press [*Cambridge History of Medicine*]

[B88] Porter TM Trust in Numbers: The Pursuit of Objectivity in Science and Public Life 1995 Princeton, NJ: Princeton University Press 10.1177/030631299029004007 11623934

[B89] Bland JM Altman DG Statistics notes: Bayesians and frequentists British Medical Journal 1998 317 1151 1160 9784463 10.1136/bmj.317.7166.1151 PMC1114120

[B90] Clarke M Chalmers I Discussion sections in reports of controlled trials published in general medical journals: islands in search of continents? The Journal of the American Medical Association 1998 280 3 280 282 10.1001/jama.280.3.280 9676682

[B91] Clarke M Alderson P Chalmers I Discussion sections in reports of controlled trials published in general medical journals The Journal of the American Medical Association 2002 287 21 2799 2801 10.1001/jama.287.21.2799 12038916

[B92] Ioannidis JPA Haidich A Lau J Any casualties in the clash of randomised and observational evidence? No - recent comparisons have studied selected questions, but we do need more data British Medical Journal 2001 322 7291 879 880 [PMC1120057] 10.1136/bmj.322.7291.879 11302887 PMC1120057

[B93] Lawlor DA Smith GD Kundu D Bruckdorfer KR Ebrahim S Those confounded vitamins: what can we learn from the differences between observational versus randomised trial evidence? Lancet 2004 363 9422 1724 1727 10.1016/S0140-6736(04)16260-0 15158637

[B94] Vandenbroucke JP When are observational studies as credible as randomised trials? Lancet 2004 363 9422 1728 1731 10.1016/S0140-6736(04)16261-2 15158638

[B95] Michiels S Koscielny S Hill C Prediction of cancer outcome with microarrays: a multiple random validation strategy Lancet 2005 365 9458 488 492 10.1016/S0140-6736(05)17866-0 15705458

[B96] Ioannidis JP Ntzani EE Trikalinos TA Contopoulos-Ioannidis DG Replication validity of genetic association studies Nature Genetics 2001 29 3 306 309 10.1038/ng749 11600885

[B97] Ioannidis JPA Contradicted and initially stronger effects in highly cited clinical research The Journal of the American Medical Association 2005 294 2 218 228 10.1001/jama.294.2.218 16014596

[B98] Colhoun HM McKeigue PM Smith GD Problems of reporting genetic associations with complex outcomes Lancet 2003 361 9360 865 872 10.1016/S0140-6736(03)12715-8 12642066

[B99] Ioannidis JPA Genetic associations: false or true? Trends in Molecular Medicine 2003 9 4 135 138 10.1016/S1471-4914(03)00030-3 12727138

[B100] Ioannidis JPA Microarrays and molecular research: noise discovery? Lancet 365 9458 454 455 15705441 10.1016/S0140-6736(05)17878-7

[B101] Neyman J Pearson ES On the problem of the most efficient tests of statistical hypotheses Philosophical Transactions of the Royal Society of London. Series A, Containing Papers of a Mathematical or Physical Character 1933 231 289 337 [ArticleType: primary article/Full publication date: 1933/Copyright © 1933 The Royal Society] 10.1098/rsta.1933.0009

[B102] Strunk A Bhalla V Clopton P Nowak RM McCord J Hollander JE Duc P Storrow AB Abraham WT Wu AHB Steg G Perez A Kazanegra R Herrmann HC Aumont MC McCullough PA Maisel A Impact of the history of congestive heart failure on the utility of B-type natriuretic peptide in the emergency diagnosis of heart failure: results from the Breathing Not Properly Multinational Study The American Journal of Medicine 2006 119 69.e1 11 http://www.ncbi.nlm.nih.gov/pubmed/16431187 10.1016/j.amjmed.2005.04.029 16431187

[B103] McCullough PA Nowak RM McCord J Hollander JE Herrmann HC Steg PG Duc P Westheim A Omland T Knudsen CW Storrow AB Abraham WT Lamba S Wu AHB Perez A Clopton P Krishnaswamy P Kazanegra R Maisel AS B-type natriuretic peptide and clinical judgment in emergency diagnosis of heart failure: analysis from Breathing Not Properly (BNP) Multinational Study Circulation 2002 106 4 416 422 10.1161/01.CIR.0000025242.79963.4C 12135939

[B104] Glimcher PW Decisions, Uncertainty, and the Brain: The Science of Neuroeconomics 2003 Cambridge, Mass: MIT Press

[B105] Barlow H Redundancy reduction revisited Network-Computation in Neural Systems 2001 12 3 241 253 11563528

[B106] Barlow H Thorpe W, Zangwill O The coding of sensory messages Current Problems in Animal Behavior 1961 Cambridge: Cambridge University Press

[B107] Barlow H Koch C, Davis JL What is the computational goal of the neocortex? Large Scale Neuronal Theories of the Brain 1994 MIT Press 1 22

[B108] Knill DC Richards W Perception as Bayesian Inference 1996 Cambridge University Press

[B109] Kahneman D Slovic P Tversky A Judgment Under Uncertainty: Heuristics and Biases 1982 Cambridge: Cambridge University Press

[B110] Elstein AS Heuristics and biases: selected errors in clinical reasoning Academic Medicine: Journal of the Association of American Medical Colleges 1999 74 7 791 794 10429587 10.1097/00001888-199907000-00012

[B111] Dolan JG Bordley DR Mushlin AI An evaluation of clinicians' subjective prior probability estimates Medical Decision Making 1986 6 4 216 223 10.1177/0272989X8600600406 3773651

[B112] Phelps MA Levitt MA Pretest probability estimates: a pitfall to the clinical utility of evidence-based medicine? Academic Emergency Medicine: Official Journal of the Society for Academic Emergency Medicine 2004 11 6 692 694 15175211

[B113] Bornstein BH Emler AC Rationality in medical decision making: a review of the literature on doctors' decision-making biases Journal of Evaluation in Clinical Practice 2001 7 2 97 107 10.1046/j.1365-2753.2001.00284.x 11489035

[B114] Dawson N Arkes H Systematic errors in medical decision making Journal of General Internal Medicine 1987 2 3 183 187 10.1007/BF02596149 3295150

[B115] Laplace PS Thérie Analytique Des Probabilités 1847 Paris: Imprimerie royale

[B116] Olshausen BA Field DJ Emergence of simple-cell receptive field properties by learning a sparse code for natural images Nature 1996 381 6583 607 609 10.1038/381607a0 8637596

[B117] Rao RPN Olshausen BA Lewicki MS Probabilistic Models of the Brain: Perception and Neural Function 2002 The MIT Press

[B118] Rieke F Spikes: Exploring the Neural Code (Computational Neuroscience) 1997 Cambridge, Mass: MIT Press

[B119] Koch C Davis JL Large-Scale Neuronal Theories of the Brain 1994 Storming Media

[B120] Oaksford M Chater N The probabilistic approach to human reasoning Trends in Cognitive Sciences 2001 5 8 349 357 10.1016/S1364-6613(00)01699-5 11477004

[B121] Baker C Tenenbaum J Saxe R Bayesian models of human action understanding Advances in Neural Information Processing Systems 2006 18 99

[B122] Griffiths TL Tenenbaum JB Optimal predictions in everyday cognition Psychological Science 2006 17 9 767 773 10.1111/j.1467-9280.2006.01780.x 16984293

[B123] Edwards AWF Likelihood 1992 Expanded Baltimore: Johns Hopkins University Press

[B124] Skellam JG Models, inference, and strategy Biometrics 1969 25 3 457 475 10.2307/2528899 5824399

[B125] Azizi F Ghanbarian A Madjid M Rahmani M Distribution of blood pressure and prevalence of hypertension in Tehran adult population: Tehran Lipid and Glucose Study (TLGS), 1999-2000 Journal of Human Hypertension 2002 16 5 305 312 10.1038/sj.jhh.1001399 12082490

[B126] Cowie MR Struthers AD Wood DA Coats AJ Thompson SG Poole-Wilson PA Sutton GC Value of natriuretic peptides in assessment of patients with possible new heart failure in primary care Lancet 1997 350 9088 1349 1353 10.1016/S0140-6736(97)06031-5 9365448

[B127] Leibniz GW Dissertatio De Arte Combinatoria, in Qua Ex Arithmeticae Fundamentis Complicationum Ac Transpositionum Doctrina Novis Praeceptis Extruitur, & Usus Ambarum Per Universum Scientiarum Orbem Ostenditur; Nova Etiam Artis Meditandi, Seu Logicae Inventionis Semina Sparguntur apud Joh. Simon Fickium et Joh Polycarp. Seuboldum, Literis Sporelianis: Lipsiae 1666

[B128] Couturat L La Logique De Leibniz Dapres Des Documents Inedits 1901 Collection historique des grands philosophes. Paris: F. Alcan

[B129] Boole G An Investigation of the Laws of Thought, on Which Are Founded the Mathematical Theories of Logic and Probabilities 1961 New York: Dover Publications

[B130] Boole G The Laws of Thought (1854) 1952 La Salle, Ill: The Open Court Pub. Co

