# Supplementary material for: Significance testing as perverse probabilistic reasoning
Source: BMC Med. 2011 Feb 28;9:20. doi: 10.1186/1741-7015-9-20 (PMC3058025; doi:10.1186/1741-7015-9-20)
Supplement: Additional file 1 — Supplemental material. [file 1741-7015-9-20-S1.PDF]

Significance Testing as Perverse Probabilistic Reasoning: Supplemental Material

M Brandon Westover*[1], Kenneth D Westover[2], Matt T Bianchi[1]

[1]Department of Neurology, Massachusetts General Hospital, Harvard Medical School, Boston, MA, USA
[2]Harvard Radiation Oncology Program, Harvard Medical School, Boston, MA, USA

Email: M Brandon Westover* - mwestover@partners.org; Kenneth D Westover - kwestover@partners.org; Matt T Bianchi - mtbianchi@partners.org;

*Corresponding author

Fundamental 'forward' and 'backward' probabilities in diagnostic testing

Interpreting diagnostic tests

When a patient 'tests positive' for a disease, what is the probability that this patient actually has the disease? For concreteness, consider using the serum level of brain natriuretic peptide (BNP) as a test for congestive heart failure, defining a positive result as BNP > 100, which reportedly has a sensitivity of 97% and specificity of 84% [1]. The interpretation of a positive result depends on the clinical context (e.g. the presence or absence of dyspnea on exertion, jugular venous distention, third heart sound, or edema), that is, on the pretest probability. The nomogram in Figure 1 allows one to precisely quantify the amount by which one's clinical suspicion (i.e. pretest probability) should be swayed by the BNP value [2].

Positive predictive values

The diagnostic testing problem can formulated in general terms as follows. Suppose we must decide whether or not patients have a particular disease. We consider two mutually exclusive hypotheses: H_1 = 'the disease is present', or H_0 = 'the disease is absent', as determined by some gold-standard (e.g. a biopsy, or observation and further testing). Suppose a test for the disease (other than the gold standard) gives results as either negative D_0 or positive D_1. Patients can be divided into four groups: true positives (H_1, D_1), false positives (H_0, D_1), true negatives (H_0, D_0), and false negatives (H_1, D_0); we denote the number in each group by TP, FP, TN, FN, respectively (see table). From these numbers we estimate eight important probabilities: four 'forward' and four 'backward' probabilities.

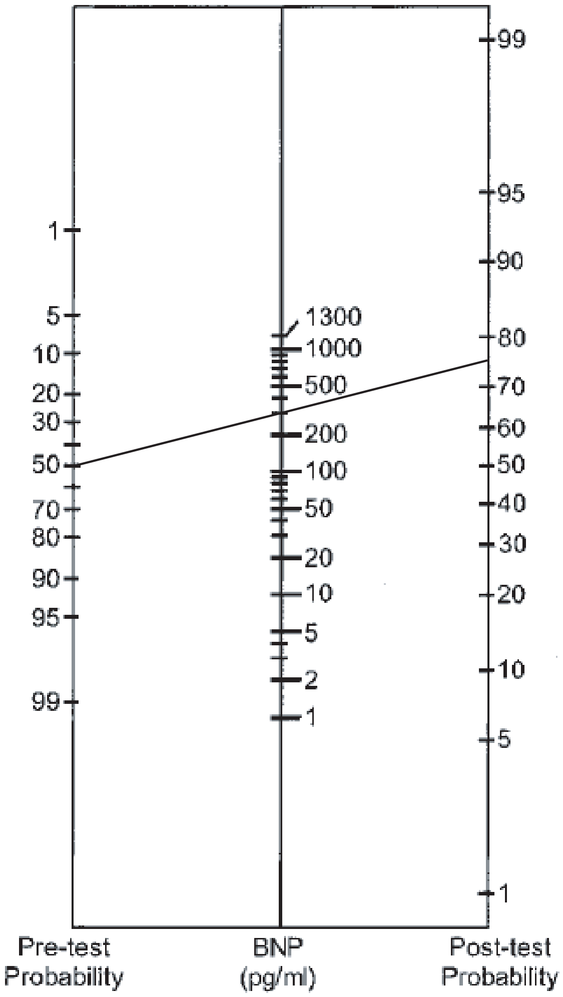

Figure 1: BNP probability nomogram. A line drawn from the pretest probability through the measured BNP yields the post test probability of CHF. In this case a BNP of 300 pg/ml in a patient where the pretest probability is 50% gives a post-test probability of 75%.

		Gold standard	
		H_1	H_0
Test results	D_1	TP	FP
	D_0	FN	TN

Four fundamental forward probabilities

The four forward probabilities are the sensitivity and specificity (a.k.a. true positive rate and true negative rate, respectively), and their complements, the false positive and false negative rates, given by the following ratios:

$$\begin{aligned} TPR &= Pr(D_1|H_1) &= TP/(TP+FN) \\ TNR &= Pr(D_0|H_0) &= TN/(TN+FP) \\ FPR &= Pr(D_1|H_0) &= FP/(FP+TN) \\ FNR &= Pr(D_0|H_1) &= FN/(FN+TP). \end{aligned}$$

Here $Pr(X|Y)$ is read 'the probability that X is true given that Y is true', or more compactly, 'the probability of X given Y'. The second variable in these expressions is called the 'conditioning variable', so that $Pr(X|Y)$ is also sometimes read as 'the probability of X conditioned on Y'.

In 'forward probabilities', the variable behind the conditioning line can often be thought of as a cause or explanation leading 'forward' to the the variable that occurs in front of the conditioning line, whose probability is expressed. For example, the presence of disease H_1 is thought of as causing positive test results D_1 in a certain proportion of these patients (or with a certain *probability* in an individual patient), and $Pr(D_1|H_1)$ thus allows one to predict in some measure whether someone with the disease will have a positive test result. Meanings and synonyms for the forward probabilities are summarized in Table 1.

Four fundamental backward probabilities

In medicine, we are usually more interested in *backward* probabilities: Typically, the fact in hand is *not* the disease status; rather, we are given some data, say a positive test result D_1, and we wish to 'go backward' from this to infer whether or the patient has the disease, 'conditioned on' the test result. These probabilities are represented by the expressions $Pr(H_0|D_1)$ and $Pr(H_1|D_1)$, respectively.

Each of the four forward probabilities has a corresponding backward probability. For example, $Pr(H_1|D_1)$, called (in the present context) the positive predictive value, is the backward probability corresponding to the sensitivity, $Pr(D_1|H_1)$; the term 'backward' refers to the swapping of the positions in the two expressions of H_1 and D_1 with

Table 1:

Fundamental forward probabilities	Fundamental backward probabilities
**True negative rate, TNR, specificity,** $Pr(D_0\|H_0)$<br>○ Fraction of ⊖ tests among healthy pts.<br>○ Probability of testing ⊖ among healthy pts.<br>○ Probability of ⊖ results when H_0 is true.<br>○1 - false positive rate	**Negative Predictive Value, NPV,** $Pr(H_0\|D_0)$<br>○ Fraction of healthy pts among those with ⊖ tests.<br>○ Probability that a ⊖ result is true.<br>○ Probability that H_0 is true when the test is ⊖.<br>○ 1 - false discovery rate
**True positive rate, TPR, sensitivity,** $Pr(D_1\|H_1)$<br>○ Fraction of diseased pts testing ⊕<br>○ Probability of testing ⊕ among diseased pts<br>○ Probability of rejecting H_0 when it is false<br>○ 1- false negative rate	**Positive Predictive Value, PPV,** $Pr(H_1\|D_1)$<br>○ Fraction of ⊕ results that are true<br>○ Probability of disease among pts who test ⊕<br>○ Probability that H_1 is true when the results are ⊕.<br>○ 1 - False Omission Rate
**False positive rate, FPR, Type I Error,** $Pr(D_1\|H_0)$<br>○ Fraction of healthy pts testing ⊕<br>○ Probability of testing ⊕ among healthy pts<br>○ Probability of rejecting H_0 when it is true<br>○1 - specificity	**False Discovery Rate, FDR,** $Pr(H_0\|D_1)$<br>○ Fraction of ⊕ results that are false<br>○ Probability of health among pts who test ⊕<br>○ Probability that H_0 is true despite ⊕ results.<br>○ 1 - Positive Predictive Value
**False negative rate, FNR, Type II Error,** $Pr(D_0\|H_1)$<br>○ Fraction of diseased pts testing ⊖<br>○ Probability of testing ⊖ among diseased pts<br>○ Probability of accepting H_0 when it is false<br>○ 1 - sensitivity	**False Omission Rate, FOM,** $Pr(H_1\|D_0)$<br>○ Fraction of ⊖ results that are false<br>○ Probability of disease among pts who test ⊖<br>○ Probability that H_0 is false despite ⊖ results.<br>○ 1 - Negative Predictive Value.

respect to the conditioning line. Similarly the negative predictive value, $Pr(H_0|D_0)$, is the backward probability for the true negative rate $Pr(D_0|H_0)$. The true and false negative rates each have a complementary probability obtained by subtracting from one: $Pr(H_1|D_0) = 1 - Pr(H_0|D_0)$ (called the 'false omission rate'), and $Pr(H_0|D_1) = 1 - Pr(H_1|D_1)$ (called the 'false discovery rate'), which are respectively the backward probabilities for the the false negative rate $Pr(D_0|H_1)$ and and false positive rate $P(D_1|H_0)$. The four backward probabilities are given by the ratios:

$$
\begin{aligned}
PPV &= Pr(H_1|D_1) &= TP/(TP + FP) \\
NPV &= Pr(H_0|D_0) &= TN/(TN + FN) \\
FDR &= Pr(H_1|D_0) &= FP/(TP + FP) \\
FOR &= Pr(H_0|D_1) &= FN/(TN + FN)
\end{aligned}
$$

The meanings and names of these probabilities are summarized in Table 1.

Cox's Theorem

> *One of the most familiar facts of our experience is this: that there* is *such a thing as common sense, which enables us to do plausible reasoning in a consistent way. People who have the same background of experience and the same amount of information about a proposition come to pretty much the same conclusions as to its plausibility...Therefore the human brain must contain some fairly definite mechanism for plausible reasoning... But in order for this to be possible, there must exist consistent rules for carrying out plausible reasoning, in terms of operations so definite that they can be programmed on the computing machine which is the human brain. -E.T. Jaynes [3]*

The quest for a quantitative 'model of thought' has occupied a distinguished list of thinkers [4], some of the most famous being Leibniz [5, 6], Boole [7, 8], and Laplace [9]. This quest began with the development of deductive logic, and ultimately led to the modern mathematical theory of probability. Remarkably, though the essential ideas of probability theory have been in existence at least since the late 1700's with the work of Laplace, it was only recently demonstrated that the laws of probability constitute the *only possible* rational system of inference. This result, known as 'Cox's theorem', was established by the work of R. T. Cox (1898-1991) [10] and E. T. Jaynes (1922-1998) [3], who showed mathematically that the laws of probability are implicit in a few generic properties that are desirable in any system of rational inference. Though the full technical proof of Cox's theorem is outside the scope of this essay, the major points are accessible and well worth understanding. Readers interested in further details are referred the excellent discussions by Jaynes [3] and more recently by Van Horn [11].

According to Cox and Jaynes, any reasonable method of plausible reasoning should at minimum meet three basic requirements:

1. Plausibility must be quantifiable.
2. Plausibility must respect common sense.
3. Plausibility must be consistent.

To explain these requirements, we first need to introduce some notation. Propositions will be symbolized by capital letters, and the negation of a proposition will be indicated by an overline, e.g. $\overline{A}$ denotes the statement 'A is not true' or simply 'not A'. We will use the symbol $\mathcal{I}$ to represent the totality of one's 'background state of knowledge', i.e. the set of propositions one holds as true, and in light of which the plausibility of other propositions is evaluated. Thus, $\mathcal{I}$ might represent the composite of one's knowledge of pathophysiology, the medical literature, personal experience, and the available data about a particular patient (e.g. test results, physical exam, past medical history).

Finally, $(A|\mathcal{I})$ will denote the plausibility we assign to proposition A given background knowledge $\mathcal{I}$. Similarly, $(A|B,\mathcal{I})$ denotes 'the plausibility of A given both $\mathcal{I}$ and that B is true'.

Note that we are temporarily using the word 'plausibility', deliberately avoiding the word 'probability', because the point of Cox's theorem is that the rules of probability theory can be *derived* from the three basic requirements of plausible reasoning. We are now in a position to explain Cox's three requirements in more detail.

Plausibility must be quantifiable.

The fact that plausibilities can be arranged in order of degree is commonplace in all everyday reasoning: One can be more confident that a flipped coin will land Heads than that a car will crash on Wednesday at a certain intersection, and more confident still that $1 + 1 = 2$. The fact that plausibility assignments can be ordered means that they can be represented numerically. That is, we can make statements like: If $(A|\mathcal{I})$ is greater than $(B|\mathcal{I})$, and $(B|\mathcal{I})$ is greater than $(C|\mathcal{I})$, then $(A|\mathcal{I}) > (C|\mathcal{I})$.

The requirement that plausibilities be quantifiable can also be motivated on physical grounds, as pointed out by Jaynes: In any physical reasoning system (e.g. a computer or a brain), the degree of plausibility assigned to a proposition ultimately corresponds to a physical quantity, like a voltage or ion concentration, which can be quantified and compared in numerical terms.

At the extreme ends of plausibility we have certain falsehood and certain truth, to which we assign numerical values F and T, respectively.[1] By convention, smaller numbers represents less plausibility, so that for any proposition A we can say $F \leq (A|\mathcal{I}) \leq T$.

Plausibility must respect common sense.

By this requirement we mean two things.

(1) Compatibility with deductive logic. Our plausibility calculus should reduce to the rules of deductive logic in cases where no uncertainty is involved, and it should remain in qualitative agreement with logic in certain basic ways under uncertain circumstances. More specifically, we impose the following requirements.

- If a proposition A is certain, then its negation cannot also be true,[2] i.e. we do not allow both $(A|\mathcal{I}) = T$ and $(\overline{A}|\mathcal{I}) = T$.
- If two statements A and B imply one another[3], then they must be equally plausible, i.e. if $A \Rightarrow B$ and $B \Rightarrow A$, then $(A|\mathcal{I}) = (B|\mathcal{I})$.

[1] By convention, we set $T = 1$ and $F = 0$.
[2] Van Horn refers to this condition as the *consistency* of $\mathcal{I}$.
[3] In logical jargon, we say that A and B are 'equivalent', denoted $A \Leftrightarrow B$, or 'A is true if and only if B is true'.

- The plausibility of a proposition that is true by logical necessity (i.e. a tautology) should not be affected by conditioning,[4] ie. if A is a tautology, then $(A|\mathcal{I}) = T$, regardless of what propositions constitute $\mathcal{I}$.
- If we are uncertain whether A is true or false, then the truth of $\overline{A}$ must also be uncertain i.e. if $F < (A|\mathcal{I}) < T$, then $F < (\overline{A}|\mathcal{I}) < T$.
- If B and C are both true, then (B, C) is true, and similarly if (B, C) is true, then B and C must be individually true.

If these statements seem obvious or too trivial to need stating, that is part of the point!

(2) Inverse relationship between negations. The plausibility of a proposition and its inverse should vary in an inverse way, such that if the plausibility $(A|\mathcal{I})$ increases, then $(\overline{A}|\mathcal{I})$ should decrease (though not necessarily by the same amount). More formally, there must be a function S such that

$$(\overline{A}|\mathcal{I}) = S[(A|\mathcal{I})],$$

where S is a monotonically decreasing function. Three examples of such functions are shown in Figure 2.

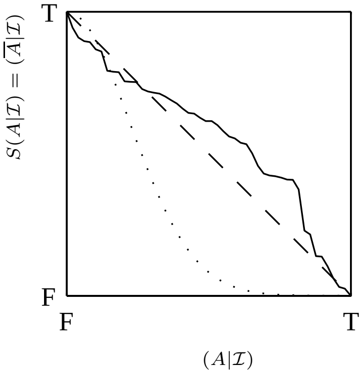

Figure 2: Figure 2. Three candidate functions for S.

Plausibility must be consistent.

If a conclusion can be reached by more than one line of reasoning, each such line of reasoning must yield the same result. Consider a case in which we ultimately conclude that a compound proposition, $(A, B, \mathcal{I})$ is true, but we reach this conclusion in sequential steps. We can either decide first that A is true, then, having accepted A, decide that B is

[4] A simple example of a tautology is the compound propositions $(A \vee \overline{A})$, which is true regardless of which proposition A actually stands for. A more complex example is the compound proposition $(A, B) \vee (\overline{A}) \vee (\overline{B})$.

true, or vice versa. That is, we can regard the reasoning from $\mathcal{I}$ to the conclusion that all three $A, B, \mathcal{I}$ are true as consisting of a sequence of transitions along two alternative routes, as depicted in the following diagram:

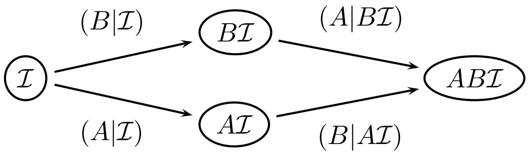

These considerations imply that, in the case of conjunctions, plausibility obeys a function F of the form [5]

$$(A, B|\mathcal{I}) = F[(B|\mathcal{I}), (A|B, \mathcal{I})] = F[(A|\mathcal{I}), (B|A, \mathcal{I})].$$

One can also arrive at this functional form for F by exhaustively listing all possible alternatives involving various combinations of A, B, AB, AI, etc, and eliminating those that lead to absurdities [11, 12].

Implications of the basic desiderata

Cox's theorem states that any system of reasoning that conforms to the preceding desiderata must obey the following rules. Let Pr be a function that quantifies the degree of plausibility. Then Cox's theorem dictates that Pr obeys the following rules:

- $0 \leq Pr(A) \leq 1$,
- $Pr(A) = 0$ when A is known to be false,
- $Pr(A) = 1$ when A is known to be true,
- $Pr(A) + Pr(\overline{A}) = 1$
- $Pr(B) = Pr(B, A) + Pr(B, \overline{A})$
- $Pr(A|B) = Pr(A, B)/Pr(B)$
- $Pr(A|B) = \frac{Pr(B|A)Pr(A)}{Pr(A)Pr(B|A)+Pr(\overline{A})Pr(B|\overline{A})}$

Note that A and B represent generic propositions; B could be taken to represent the 'background knowledge' $\mathcal{I}$ if desired. These formulas constitute the laws of probability theory. These rules are not a recent invention; they been accepted by many scholars as a normative model for rationality that extends and completes deductive logic at least since the work of Laplace in the 1700's [13]. However, only recently, through the work of Cox and Jaynes, has there been a compelling case that that rational inference *must* conform to probability theory.

[5]This graphical way of motivating the functional form of F is from an early draft of Jaynes' book [12], in which Jaynes credits this argument to Alfred S. Gilman.

Explanation of the laws of probability

In this section we review the meaning of the basic laws of probability. We will borrow freely from each of the various interpretations (frequentist, set-theoretic, and Bayesian) in an effort to make the formulas intuitively transparent.

Rule 1: $0 \leq Pr(A) \leq 1$, where $Pr(A) = 0$ when A is false, and $Pr(A) = 1$ when A is true.

This rule is essentially self explanatory: $Pr(A) = 1$ means 'The truth of A is certain' or 'A is true', and $Pr(A) = 0$ means 'The falsehood of A is certain' or 'A is false'. Similarly, in frequentist terms we might say $Pr(A) = 1$ means 'A is always the case' or 'A is true for 100% of the population', and $Pr(A) = 0$ means 'A is never the case' or 'A is true of 0% of the population.' In terms of Venn diagram (Figure 3 (a)), the fraction of the large circle occupied by A can be no larger than 1, and no smaller than 0.

Rule 2: $Pr(A) + Pr(\overline{A}) = 1$

Rule 2 reflects the logical fact that at least one of any two mutually exclusive and jointly exhaustive claims must be true. That is, whenever one says something to the effect, 'either A or not A is true', we may be certain that this is correct. In terms of Venn diagrams (Figure 3(a) the large circle represents the set of all possible events of interest. The subregions A and $\overline{A}$ represent the frequencies with which these events occur.

For example, we might split a patient population into those who do (A) and do not $(\overline{A})$ have diabetes. The probability that patients either do or do not have diabetes is 1: everyone (100% of the population) must fall into one or the other category. In a population of 951 patients of whom 236 have diabetes and 715 do not, the relative proportions would be $Pr(A) = 236/951 = 0.25$ and $Pr(\overline{A}) = 715/951 = 0.75$, so

$Pr(A) + Pr(\overline{A}) = 236/951 + 715/951 = 0.25 + 0.75 = 1 = 100\%$. The same types of statements apply if we consider the probability of diabetes in a single person, in which case we break the universe of possibilities into the mutually exclusive and jointly exhaustive statements A = 'the patient has diabetes' or $\overline{A}$= 'the patient does not have diabetes'.

Of course, the universe of interest need not be binary. If we choose instead to carve the universe into n nonoverlapping regions $A_1, A_2, ..., A_n$, then Rule 2 becomes $Pr(A_1) + Pr(A_2) + ... + Pr(A_n) = 1$.

Rule 3: $Pr(B) = Pr(B, A) + Pr(B, \overline{A})$

Consider a second division of the universe of interest into categories B and $\overline{B}$, such as the division of a population into those who do (B) and do not ($\overline{B}$) have coronary artery disease (CAD), or statements that an individual is in either of two states, B= 'has CAD', or $\overline{B}$='does not have CAD'. With this second division we now have four possible groups or categories: (A, B), $(A, \overline{B})$, $(\overline{A}, B)$, and $(\overline{A}, \overline{B})$. Suppose that among our 951 patients there are 162 with diabetes and CAD (A, B), 74 with diabetes and no CAD $(A, \overline{B})$, 284 with CAD but no diabetes $(\overline{A}, B)$, and 431 with neither hypertension nor CAD. The proportions then come out to $Pr(A, B) = 162/951 = 17\%$,

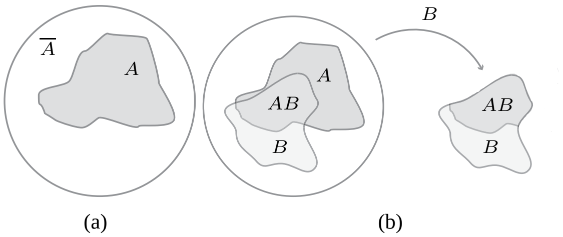

Figure 3: (a) Venn diagram for Rule 1. (b) Venn diagrams for Rules 2-4.

$Pr(A, \overline{B}) = 74/951 = 8\%$, $Pr(\overline{A}, B) = 284/951 = 30\%$, and $Pr(\overline{A}, \overline{B}) = 431/951 = 45\%$, which add up to 100%. These proportions are represented by the areas of overlap in the Venn diagram in Figure 3(b).

Now suppose we want to factor out one of the variables to find out the fraction of the population in category B, regardless of where these patients fall with respect to the A vs. $\overline{A}$ division. That is, we want to know what fraction has hypertension, regardless of diabetes status. The hypertension group is made up of some who do and some who don't have CAD, so the fraction $Pr(B)$ is the sum of $Pr(A, B)$ and $Pr(\overline{A}, B)$, or

$Pr(B) = Pr(A, B) + Pr(A, \overline{B}) = 162/951 + 74/951 = 0.17 + 0.08 = 25\%$. In the Venn diagram, $Pr(B)$ corresponds to the sum of the areas of intersection between (B, A) and $(B, \overline{A})$.

Rule 4: $Pr(A|B) = Pr(A, B)/Pr(B)$

The conditional probability $Pr(A|B)$ represents the plausibility or degree of belief that a rational agent should assign to A, having learned that B is the case. In other words, it represents the degree to which B implies A.

Many find conditional probability a difficult concept, so we will explain this rule several different ways. In frequentist terms, $Pr(A|B)$ represents the frequency with which event A occurs in circumstances where B has already been observed to be true. In terms of fractions, $Pr(A|B)$ represents the fraction of A within B. Intuitively, $Pr(A|B)$ is closely related to the quantity $Pr(A, B)$, but the two quantities are not the same. The joint probability $Pr(A, B)$ is the fraction of events that fall into both classes A and B within the set of *all* events, i.e. the number of events with properties A and B normalized by (divided by) the total number of all things, whereas $Pr(A|B)$ represents the number of things with properties A and B normalized by the number of things in B alone. In concrete terms, $Pr(A, B)$ represents the fraction of patients with both diabetes and CAD within the entire population of 951 patients, $Pr(A, B) = 162/951 = 17\%$, whereas the conditional probability $Pr(A|B)$ is the fraction of patients with diabetes and CAD within the subpopulation of 446 patients who have CAD, $Pr(A|B) = 162/446 = 36\%$.[6] Finally, in terms of Venn diagrams, $Pr(A, B)$ is the fractional area of (A, B) within the large circle, whereas when we

[6]It might be more clear to write $Pr(A|B)$ as $Pr(A, B|B)$, but this is usually not done, because it is redundant to say 'the probability of A and B given B'.

condition on B, the universe effectively collapses to include only B, and $Pr(A|B)$ is thus the fraction of the total area of B occupied by A. Mathematically, this constriction corresponds to dividing the fraction $Pr(A, B)$ by $Pr(B)$. As a sanity check, one can verify this in the extreme case in which all of B is occupied by A, i.e.

$Pr(A, B) = Pr(B)$, in which case $Pr(A|B) = 1$.

Rule 5 (Bayes' rule)

Bayes' rule follows from the preceding rules by a sequence of simple substitutions. In the following, the rule being used is noted above the equals sign:

$$\begin{aligned} Pr(A|B) &\overset{4}{=} \frac{Pr(A,B)}{Pr(B)} \\ &\overset{4}{=} \frac{Pr(B|A)Pr(A)}{Pr(B)} \\ &\overset{3}{=} \frac{Pr(B|A)Pr(A)}{Pr(B,A) + Pr(B,\overline{A})} \\ &\overset{4}{=} \frac{Pr(B|A)Pr(A)}{Pr(B|A)Pr(A) + Pr(B|\overline{A})Pr(\overline{A})}, \end{aligned}$$

While mathematically straightforward, this derivation provides little insight into the meaning or behavior of Bayes' rule. The interpretation and mechanics of using Bayes' rule are discussed in the main text.

1 Additional details of p-value calculations for coin flipping examples

Here we reproduce for the interested reader a few additional technical details about the calculation of p-values for the coin flipping example discussed in the discussion of p-values, for the subsection titled 'Angle 2. p-values for coin flipping experiments'.

Let us carry out the p-value calculation in detail for a simple coin flipping experiment, where we wish to decide whether a coin is fair (equal probability of heads or tails) or biased (unequal probabilities). Note that the p-value in this case is 'two-sided'. Following the NHSTP:

1. Let H_0= 'the probability of heads is $1/2$', H_1= 'probability of heads $\neq 1/2$'.

2. The experiment will consist of flipping a coin a number of times n, and the data D will thus be a series of heads or tails. For our test statistic T, let us compute the difference between 1/2 and the fraction of heads, i.e. if k of the n coin tosses land as heads, then $d = T(D) = |1/2 - k/n|$. For this example, let us put $n = 10$.

3. We set the significance level to the conventional value $\alpha = 0.05 = 5\%$.

4. Having done the experiment suppose we get data $D = (H, H, H, H, H, H, T, H, H, T)$. This sequence contains eight heads, so $d = T(D) = |1/2 - 8/10| = 0.3$.

5. To calculate the p-value, we must consider all the ways in which the data could have been as extreme or more extreme than observed, assuming that the null hypothesis is true. That is, we need to consider all possible outcomes for the data D such that $T(D) \geq 0.3$, and calculate the joint probability of these outcomes, assuming that the coin is fair. Clearly, observing 8, 9, or 10 heads would be 'as extreme or more extreme' than our result of 8 heads. Since the null hypothesis assumes equal probability for heads and tails, symmetry dictates that observing 0, 1, or 2 heads would also qualify. Hence, the p-value is

$$\begin{aligned} p &= Pr(T(D) \geq 0.2 | H_0) \\ &= Pr(k \geq 8 \text{ or } k \leq 2 | H_0). \end{aligned}$$

The probability $p_n(k)$ of obtaining k heads in a series of n fair coin flips follows a binomial distribution

$$p_n(k) = C_k^n \, (1/2)^n \, ,$$

where $C_k^n = \frac{n!}{k!(n-k)!}$ (the binomial coefficient), so in this case, with $n = 10$ and $k = 8$, the p-value is:

$$\begin{aligned} p &= (1/2)^n \, [C_0^{10} + C_1^{10} + C_2^{10} + C_8^{10} + C_9^{10} + C_{10}^{10}] \\ &= 10.94\%. \end{aligned}$$

6. Since $p \geq 5\%$, the NHSTP tells us to accept the null hypothesis, concluding that the coin is fair.

Before leaving this example, it is instructive to examine its associated Type I and II error rates. The Type I error rate (false positive rate) in this case is the probability of incorrectly declaring the coin unfair (H_1) when in fact it is fair (H_0), that is, the probability of getting $p \leq \alpha$ when in fact H_0 is true. It turns out that had we observed just one more head then the NHSTP would have declared a positive result. That is, suppose $k = 9$, or $d = T(D) = |1/2 - 9/10| = 0.4$. Then

$$\begin{aligned} p &= Pr(d \geq 0.4 | H_0) \\ &= Pr(k \geq 9 \text{ or } k \leq 1 | H_0). \\ &= (1/2)^n \, [C_0^{10} + C_1^{10} + C_9^{10} + C_{10}^{10}] \\ &= 2.15\% \end{aligned}$$

Thus, we see that $p \leq \alpha$ whenever $d \geq 0.4$, hence the Type I error rate or false positive rate is

$$FPR = Pr(D_1 | H_0) = Pr(p \leq \alpha | H_0) = 2.15\%.$$

Calculation of the false negative rate requires additional assumptions, because a coin can be biased in many (in fact, infinitely many) ways. Perhaps the least committed alternative hypothesis H_1 is that for biased coins *any* heads probability different from 1/2 is equally likely. In this case the false negative rate turns out to be [7]

$$\begin{aligned} FNR &= Pr(D_0|H_1) = \sum_{k=3}^{8} C_k^n \int_0^1 q^k (1-q)^{N-k} dq \\ &= 72.73\%. \end{aligned}$$

References

1. M R Cowie, A D Struthers, D A Wood, A J Coats, S G Thompson, P A Poole-Wilson, and G C Sutton. Value of natriuretic peptides in assessment of patients with possible new heart failure in primary care. *Lancet*, 350(9088):1349–53, November 1997.
2. Peter A McCullough, Richard M Nowak, James McCord, Judd E Hollander, Howard C Herrmann, Philippe G Steg, Philippe Duc, Arne Westheim, Torbjørn Omland, Cathrine Wold Knudsen, Alan B Storrow, William T Abraham, Sumant Lamba, Alan H B Wu, Alberto Perez, Paul Clopton, Padma Krishnaswamy, Radmila Kazanegra, and Alan S Maisel. B-type natriuretic peptide and clinical judgment in emergency diagnosis of heart failure: analysis from breathing not properly (BNP) multinational study. *Circulation*, 106(4):416–22, July 2002.
3. Edward T. Jaynes. How does the brain do plausible reasoning? In G. J. Erickson and C. R. Smith, editors, *Maximum-Entropy and Bayesian Methods in Science and Engineering*. Kluwer Academic Publishers, 1988.
4. D. Mumford. The dawning of the age of stochasticity. *Mathematics: Frontiers and Perspectives*, page 197–218, 2000.
5. Gottfried Wilhelm Leibniz. *Dissertatio De Arte Combinatoria, in Qua Ex Arithmeticae Fundamentis Complicationum Ac Transpositionum Doctrina Novis Praeceptis Extruitur, & Usus Ambarum Per Universum Scientiarum Orbem Ostenditur; Nova Etiam Artis Meditandi, Seu Logicae Inventionis Semina Sparguntur*. apud Joh. Simon Fickium et Joh. Polycarp. Seuboldum, Literis Spörelianis, Lipsiae, 1666.
6. Louis Couturat. *La Logique De Leibniz D'après Des Documents Inédits*. Collection historique des grands philosophes. F. Alcan, Paris, 1901.
7. George Boole. *An Investigation of the Laws of Thought, on Which Are Founded the Mathematical Theories of Logic and Probabilities*. Dover Publications, New York, 1961.
8. George Boole. *The Laws of Thought (1854)*. The Open Court Pub. Co, La Salle, Ill, 1952.
9. Pierre Simon Laplace. *Théorie Analytique Des Probabilités*. Imprimerie royale, [Paris, 1847.
10. Richard Threlkeld Cox. *The Algebra of Probable Inference*. Johns Hopkins Press, Baltimore, 1961.
11. Kevin S Van Horn. Constructing a logic of plausible inference: a guide to cox's theorem. *International Journal of Approximate Reasoning*, 34:3—24, 2003.
12. E. T Jaynes and G. Larry Bretthorst. *Probability Theory: The Logic of Science*. Cambridge University Press, Cambridge, UK, 2003.
13. M. Oaksford and Nick Chater. *Bayesian Rationality: The Probabilistic Approach to Human Reasoning*. Oxford cognitive science series. Oxford University Press, Oxford, 2007.
14. David J. C. MacKay. *Information Theory, Inference & Learning Algorithms*. Cambridge University Press, 1st edition, June 2002.

[7] The derivation of this result is beyond the scope of this paper. We refer the reader interested in the details of the calculation to Chapter 3 of [14].
